# Anti-Inflammatory Screening and Molecular Modeling of Some Novel Coumarin Derivatives

**DOI:** 10.3390/molecules20045374

**Published:** 2015-03-26

**Authors:** Radwan El-Haggar, Reem I. Al-Wabli

**Affiliations:** 1 Department of Pharmaceutical Chemistry, Faculty of Pharmacy, Helwan University, Ain Helwan, Cairo 11790, Egypt; E-Mail: radwanelhaggar@yahoo.com; 2 Department of Pharmaceutical Chemistry, College of Pharmacy, King Saud University, Riyadh 11451, Saudi Arabia

**Keywords:** 6-amino-8-hydroxycoumarin, anti-inflammatory, molecular docking

## Abstract

Coumarin and their derivatives have drawn much attention in the pharmacological and pharmaceutical fields due to their broad range and diverse biological activities. In the present work, starting from the 6-amino-7-hydroxy-4-methyl-*2H*-chromen-2-one, a series of 6-(substituted benzylamino)-7-hydroxy-4-methyl-*2H*-chromen-2-ones **1**–**11** was synthesized and assessed for their anti-inflammatory activity using the carrageenan-induced hind paw edema method. Compounds **2**, **3**, **4** and **9** showed significant (*p* < 0.001) reduction of rat paw edema volume after 1 h from the administration of the carrageenan compared to the reference drug, indomethacin. On the other hand, compounds **4** and **8** showed the highest anti-inflammatory activity, surpassing indomethacin after 3 h with 44.05% and 38.10% inhibition, respectively. Additionally, a molecular docking study was performed against the COX enzyme using the MOE 10.2010 software.

## 1. Introduction

Inflammation is a complex host response to tissue injury that is contolled by many mediators among which are the prostaglandins (PGs). Cyclooxygenase (COX) enzymes catalyse the synthesis of PGs from arachidonic acid (AA). It exists in two isoforms, a constitutive form COX-1 that has a cytoprotective role in the gastrointestinal tract and an inducible form COX-2 which is responsible for the elevated production of PGs during inflammation. Most of the non-steroidal anti-inflammatory drugs (NSAIDs) inhibit both COX-1 and COX-2 at their therapeutic doses. Compelling evidence suggests that inhibition of prostanoids produced by COX-2 can be ascribed to the anti-inflammatory, analgesic and antipyretic effects of NSAIDs. Because of that it is considered a potential target for the treatment of inflammation [1] and the design of selective COX-2 inhibitors should provide relief from the symptoms of inflammation and pain.

**Scheme 1:**
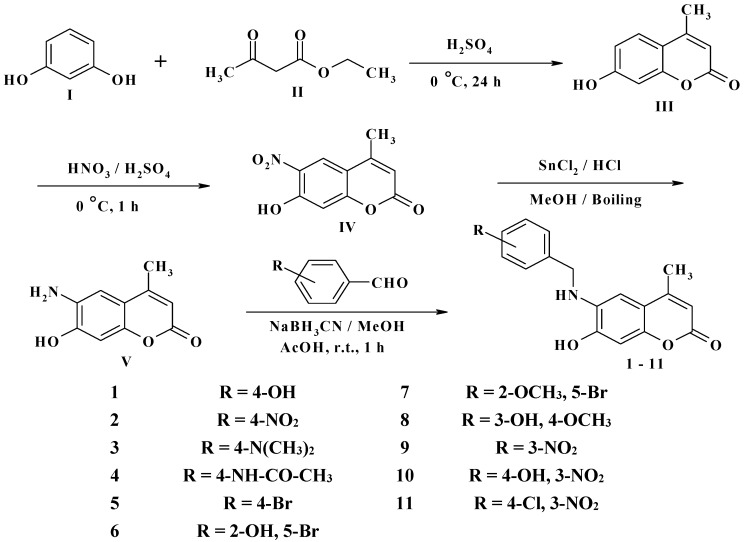
Synthesis of 6-(substituted benzylamino)-7-hydroxy-4-methyl-*2H*-chromen-2-one derivatives **1–11**.

Coumarin (*2H*-1-benzopyran-2-one) and its analogs comprise a very large class of compounds which are naturally found in plants [2]. Coumarins have attracted considerable attention due to their wide spectrum of pharmacological and biological activities as anti-coagulant, antitumor, antifungal, antiviral, antibacterial and anti-inflammatory agents [3]. Coumarins represent the core structure for many pharmaceutical compounds which have beneficial effects on human health [4,5]. Furthermore, it has been already reported that coumarin is a potential nucleus for the development of anti-inflammatory drugs [6,7,8,9,10,11]. Its hydroxyaromatic derivatives (5- or 6- or 7-hydroxycoumarin) show even more potent anti-inflammatory activity [12]. Motivated by the attempt to discover a new coumarin series with improved potency and COX-2 selectivity, we designed and synthesized some new 6-(substituted benzylamino)-7-hydroxy-4-methylcoumarin derivatives **1**–**11** (Scheme 1). The anti-inflammatory effect of the newly synthesized compounds and a reference drug (indomethacin) was evaluated by the carrageenan-induced paw edema method [13].

Furthermore, analysis of the X-ray crystal structure of AA with the COX-2 enzyme revealed that the carboxylate of the ligand coordinates with Ser530 and Tyr385 [14]. NSAIDs like indomethacin interacts with the COX-2 enzyme by forming hydrogen bonds between a carboxylic acid and Arg120, a carbonyl oxygen with Ser530 and the OH of a carboxylic acid with Tyr355 [15]. In this study, molecular docking was used to gain insight into the possible interactions of our newly synthesized compounds with the COX-1 and COX-2 enzymes.

## 2. Results and Discussion

### 2.1. Chemistry

The novel target 6-(substituted benzylamino)-7-hydroxy-4-methyl-*2H*-chromen-2-one derivatives **1**–**11** were synthesized in four steps (Scheme 1). Initially, 7-hydroxy-4-methyl-*2H*-chromen-2-one **III** was prepared via a Pechmann reaction [16] by reacting resorcinol **I** and ethyl acetoacetate **II**. Subsequently, nitration of 4-methyl-7-hydroxycoumarin **III** with a mixture of nitric acid and sulfuric acid produced a separable mixture of both 6- and 8-nitrocoumarin derivatives [17]. Several methods have been reported to reduce *o*-nitrohydroxy-4-methylcoumarins using different reducing agents [18]. In the present study, 6-amino-7-hydroxy-4-methylcoumarin **V** was obtained by reduction of 7-hydroxy-4-methyl-6-nitrocoumarin **IV** using stannous chloride in hydrochloric acid and ethanol to obtain a better yield. Finally, the target compounds **1**–**11** were obtained via reductive amination of compound **V** with the appropriate aromatic aldehyde using sodium cyanoborohydride in methanol and acetic acid (Scheme 1).

### 2.2. Anti-Inflammatory Activity

In the present investigation, the *in vivo* anti-inflammatory activity was evaluated for all the newly synthesized compounds **1**–**11** using the carrageenan-induced rat paw edema protocol. The paw edema volume was evaluated 1, 2 and 3 h after the induction of inflammation. The anti-inflammatory activity of the tested compounds and reference drug (indomethacin) were determined as the increase in paw edema volume and the results are summarized in Table 1 and as percentage inhibition (% inhibition) and summarized in Table 2. Results were expressed as the mean ± SE difference between control and treated animals using one way analysis of variance (ANOVA), followed by a Tukey-Kramer test for multiple comparisons.

In general, the data listed in Table 1 indicate that all of the newly synthesized compounds significantly (*p* < 0.001) reduce the rat paw edema volume 3 h after administration of the carrageenan compared to the reference drug, indomethacin. Compounds **2**, **3**, **4** and **9** showed a remarkable reduction of rat paw edema volume 1 h after administration of the carrageenan compared to the reference drug, indomethacin. All the tested compounds except **1**, **7** and **10** showed significant (*p* < 0.01–0.001) reduction of rat paw edema volume 2 h after the administration of the carrageenan compared to the reference drug, indomethacin.

**Table 1:** The anti-inflammatory activity of the tested compounds and reference drug (Indomethacin) in carrageenan-induced rat paw edema assay, Values are expressed as mean ± SEM, (*n* = 8).

Groups	Increase in Paw Volume (Edema Volume) (mL)			
	0 h	1 h	2 h	3 h
Control	0.42 ± 0.01	0.71 ± 0.01	0.8 ± 0.01	0.84 ± 0.01
Indomethacin (10 mg/kg)	0.45 ± 0.01	0.65 ± 0.02	0.60 ± 0.02 ***	0.56 ± 0.02 ***
1	0.44 ± 0.03	0.66 ± 0.03	0.72 ± 0.03	0.64 ± 0.03 ***
2	0.43 ± 0.02	0.58 ± 0.02 **	0.68 ± 0.02 **	0.61 ± 0.02 ***
3	0.46 ± 0.02	0.56 ± 0.02 ***	0.66 ± 0.02 ***	0.57 ± 0.02 ***
4	0.40 ± 0.02	0.50 ± 0.02 ***	0.57 ± 0.02 ***	0.47 ± 0.02 ***
5	0.46 ± 0.03	0.63 ± 0.03	0.70 ± 0.03 **	0.60 ± 0.03 ***
6	0.41 ± 0.02	0.64 ± 0.02	0.68 ± 0.02 **	0.58 ± 0.02 ***
7	0.43 ± 0.03	0.68 ± 0.03	0.72 ± 0.03	0.60 ± 0.03 ***
8	0.47 ± 0.01	0.65 ± 0.01	0.58 ± 0.01 ***	0.52 ± 0.01 ***
9	0.43 ± 0.01	0.59 ± 0.01 **	0.63 ± 0.01 ***	0.66 ± 0.02 ***
10	0.48 ± 0.03	0.61 ± 0.03	0.76 ± 0.03	0.64 ± 0.03 ***
11	0.42 ± 0.01	0.66 ± 0.01	0.63 ± 0.01 ***	0.57 ± 0.01 ***

** Significant difference at *p* < 0.01 and *** Significant difference at *p* < 0.001.

**Figure 1:**
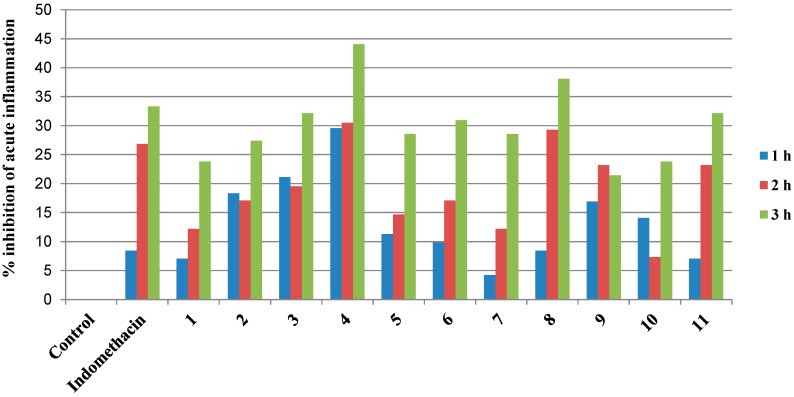
% Inhibition of acute inflammation (carrageenan-induced paw edema), (*n* = 8).

In Table 2, compounds **4** and **8** revealed higher anti-inflammatory activity that exceed the activity of indomethacin itself with 30.49% and 29.27% inhibition, respectively, at 2 h and 44.05% and 38.10% inhibition, respectively, at 3 h. In addition, compounds **3**, **11** and **6** exhibited similar anti-inflammatory activity to indomethacin at 3 h with 32.14%, 32.14% and 30.95% inhibition, respectively. On the other hand, compounds **5**, **7** and **2** showed a slightly lower anti-inflammatory activity than indomethacin at 3 h with 28.57%, 28.57% and 27.38% inhibition, respectively. Moreover, the lowest activities were measured for compounds **1**, **10** and **9** at 3 h with 23.81%, 23.81% and 21.43%, inhibition, respectively (Figure 1).

**Table 2:** % Inhibition of acute inflammation (carrageenan-induced paw edema), (*n* = 8).

Groups	% Inhibition of Acute Inflammation		
	1 h	2 h	3 h
Control	0	0	0
Indomethacin (10 mg/kg)	8.45	26.83	33.33
1	7.04	12.20	23.81
2	18.31	17.07	27.38
3	21.13	19.51	32.14
4	29.58	30.49	44.05
5	11.27	14.64	28.57
6	9.86	17.07	30.95
7	4.23	12.20	28.57
8	8.45	29.27	38.10
9	16.90	23.17	21.43
10	14.08	7.32	23.81
11	7.04	23.17	32.14

### 2.3. Molecular Docking Study

Molecular docking is used to predict the intermolecular complex formed between the compounds and the receptor or the enzyme and it gives good indication of the possible biological activities. It also predicts the strength of the binding, the energy of the complex and calculates the binding affinity using scoring functions.

The X-ray crystallographic structures of COX-1 (PDB: 1PRH) and COX-2 complexed with a non-selective inhibitor, flurbiprofen (PDB: 3PGH) were obtained from the Protein Data Bank through the internet. X-ray diffraction methods were used to solve crystal structures of both COX-1 and COX-2 with different ligands docked in their active site. Overall differences between the two enzymes structures are small. The two enzymes are highly homologous, exhibiting 61% sequence identity that reaches 87% when only the subsets of residues located in COX active site are compared. The active sites of COX-1 and COX-2 are very similar, except for two residues: Ile 523 and Val523.

Inspection of the COX active site revealed three different regions: a hydrophobic pocket beneath the heme group, defined by the residues Phe381, Tyr385, Trp387, Phe518, Ala201, Tyr248 and Leu352. The mouth of the active site, with three hydrophilic residues flanking its entrance: Arg120, Glu524, and Tyr355. These residues are arranged to form a hydrogen bond network. A larger side pocket in COX-2, defined by several conserved residues including His90 and the non-conserved residues His/Arg513 and Ile/Val523. All the tested compounds **1**–**11** showed non-selective inhibition of both COX-1 and COX-2 enzymes. Results of their interaction energies with COX-1 and COX-2 are shown in Table 3.

**Table 3:** Interaction energies of compounds **1**–**11** with the COX-1 and COX-2 enzymes.

Compound No.	Chemical Structure	dG (COX-1) Kcal/mole	dG(COX-2) Kcal/mole
Reference	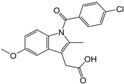	−11.5716	−11.1006
**1**	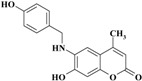	−12.2721	−10.5499
**2**	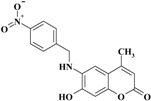	−11.5564	−10.7125
**3**	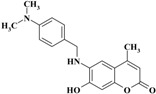	−11.1199	−10.9148
**4**	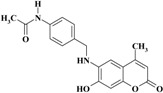	−13.5408	−11.2977
**5**	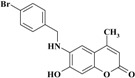	−11.3127	−11.1787
**6**	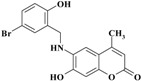	−11.5776	−10.7572
**7**	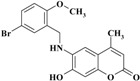	−11.0632	−10.9992
**8**	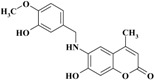	−12.4821	−12.2774
**9**	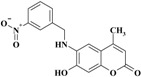	−11.3067	−10.4561
**10**	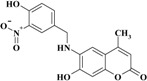	−11.7976	−11.2097
**11**	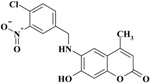	−11.3457	−10.8248

The data in Table 3 shows a rough correlation between the binding free energy ΔG values of the target compounds and their activity.

#### 2.3.1. Binding Modes of Different Compounds with COX-2 Enzyme

**Figure 2:**
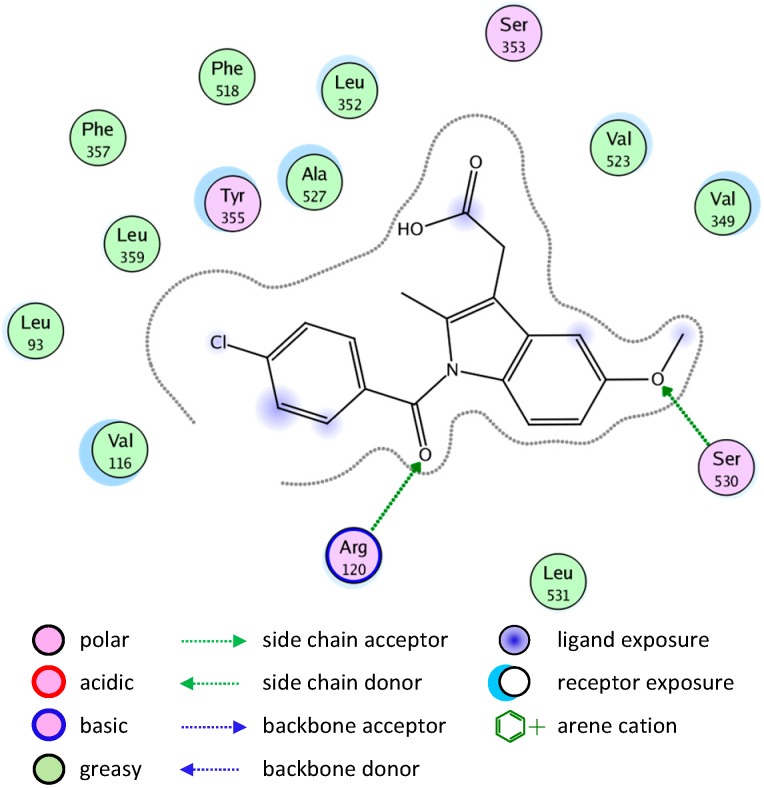
Docking of indomethacin (a non-selective inhibitor of cyclooxygenase COX-1 and 2) into the COX-2 active site.

**Figure 3:**
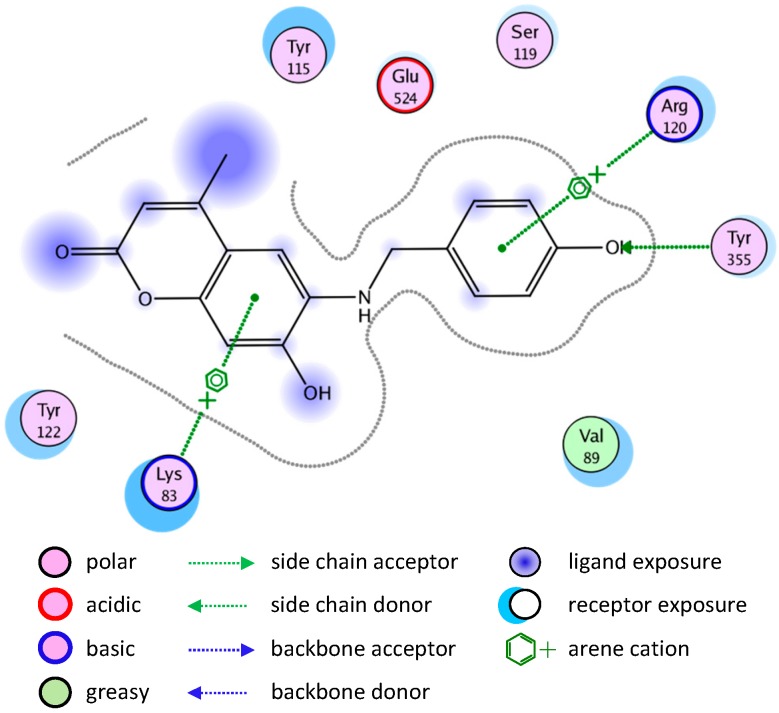
2D representation of docking of compound **1** into the COX-2 active site.

Most of the newly synthesized compounds simulate indomethacin’s binding with Arg120 and Tyr355 at its active site (Figure 2). Molecular docking of compound **1** into COX-2 active site (Figure 3) revealed several molecular interactions considered to be responsible for the observed affinity: (i) hydrogen bond interaction between the hydroxyl group of the ligand and Tyr355. (ii) Arene-cation interaction between the benzene rings of the ligand and Arg120 and Lys83.

**Figure 4:**
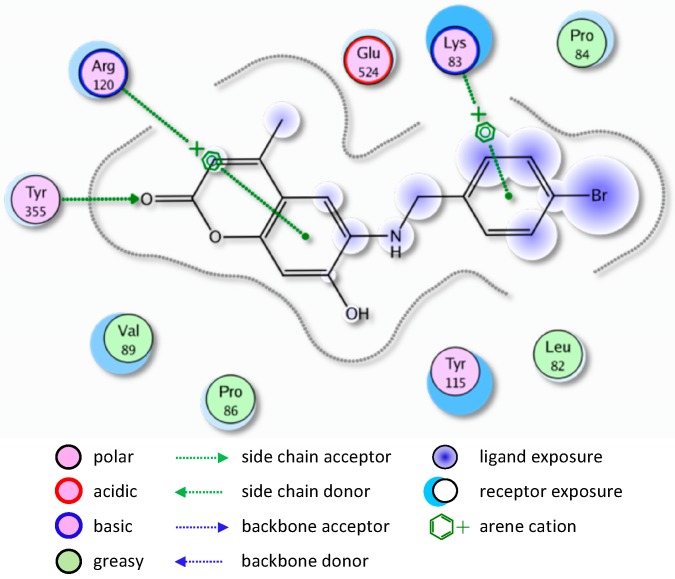
2D representation of docking of compound **5** into the COX-2 active site.

A similar binding mode was shown with compound **5** (Figure 4).

**Figure 5:**
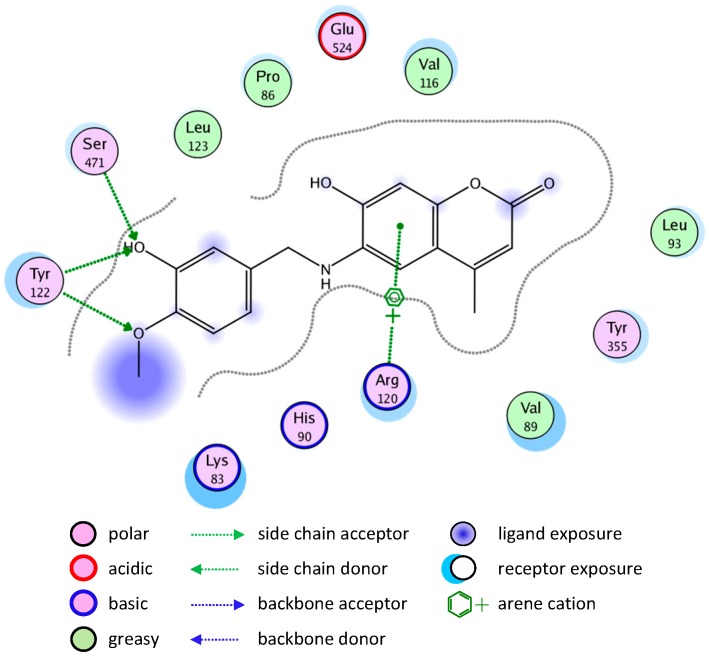
2D representation of docking of compound **8** into the COX-2 active site.

**Figure 6:**
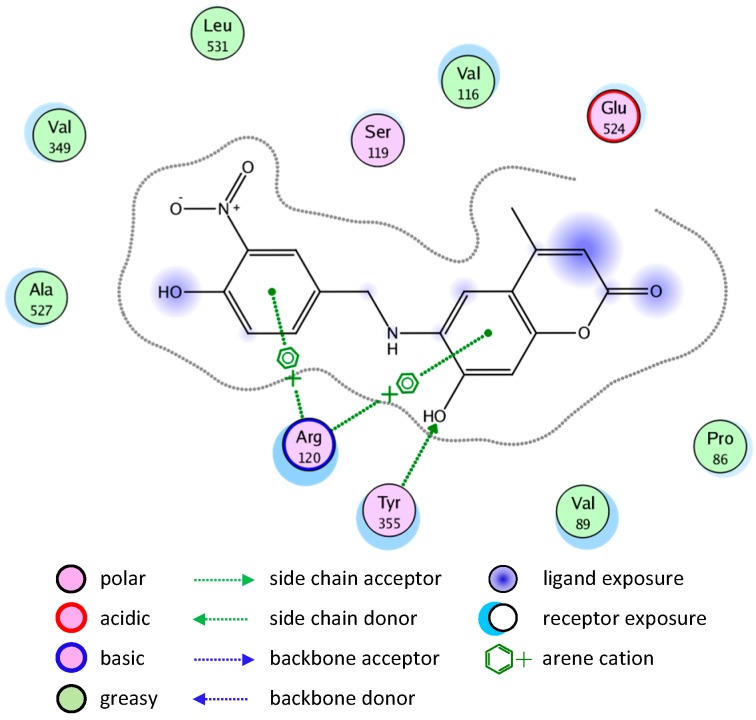
2D representation of docking of compound **10** into the COX-2 active site.

The binding of compounds **8** and **10** is shown in Figure 5 and Figure 6, respectively.

#### 2.3.2. Binding Modes of Different Compounds with the COX-1 Enzyme

**Figure 7:**
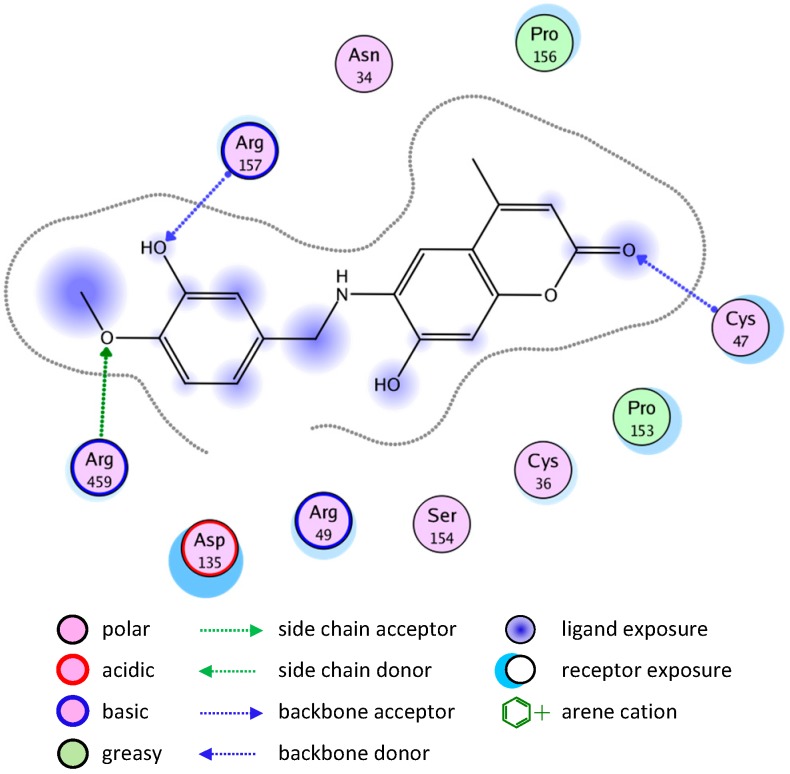
2D representation of docking of compound **8** into the COX-1 active site.

Molecular docking simulation of compound **8** into the COX-1 active site (Figure 7) revealed several molecular interactions which are considered to be responsible for the observed affinity. There are three hydrogen bond interactions: H-bond between the carbonyl group of the ligand and Cys47. The second between the O in the methoxy group of the ligand and Arg459. The third between the OH group and Arg157.

**Figure 8:**
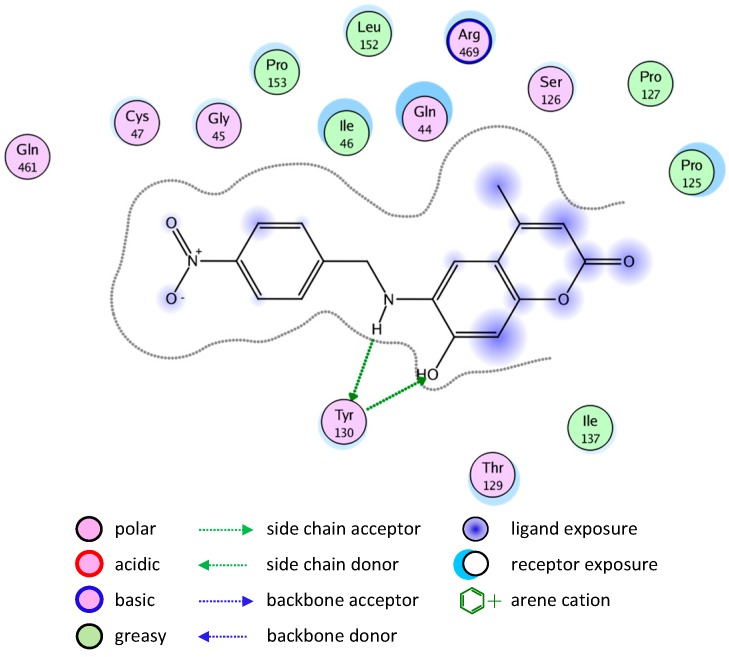
2D representation of docking of compound **2** into the COX-1 active site.

A similar binding mode was shown with compound **2** (Figure 8).

**Figure 9:**
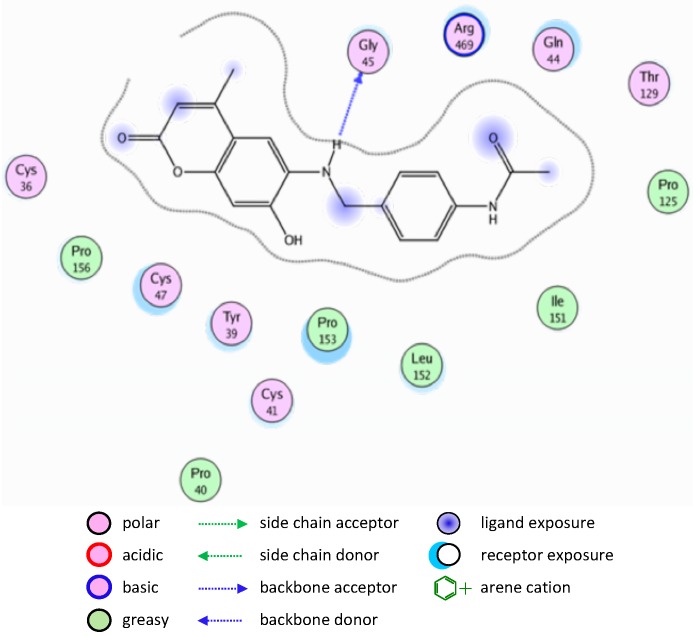
2D representation of docking of compound **4** into the COX-1 active site.

**Figure 10:**
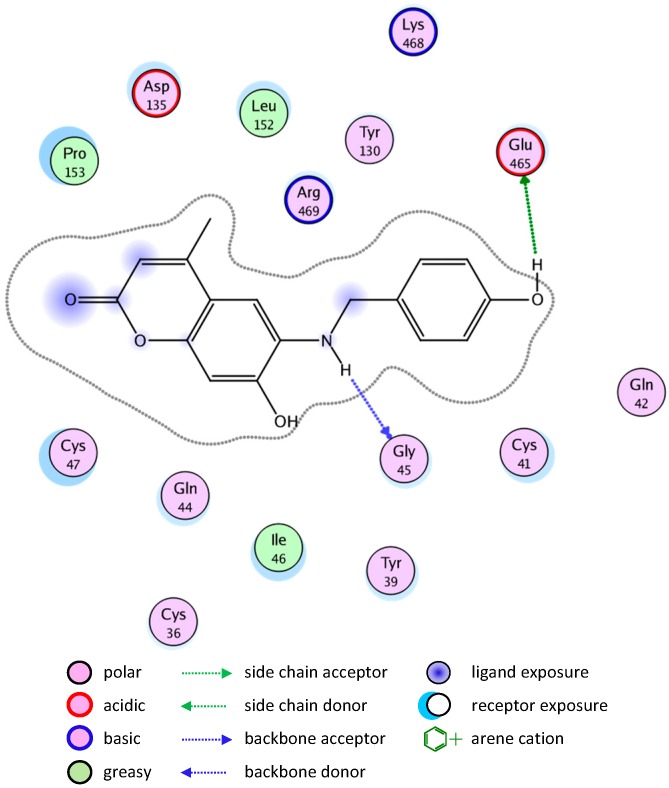
2D representation of docking of compound **1** into the COX-1 active site.

**Figure 11:**
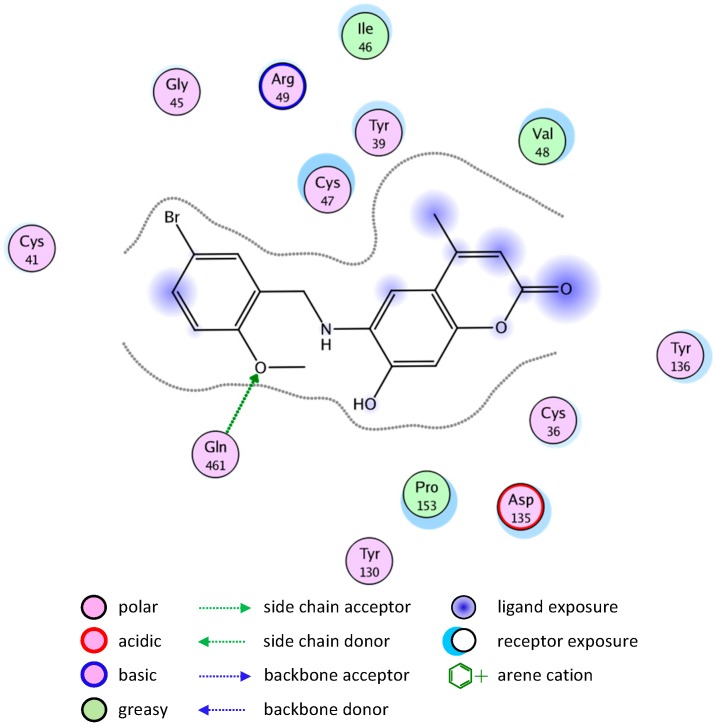
2D representation of docking of compound **7** into the COX-1 active site.

The good binding interaction of compound **4** (ΔG: −13.5408) with COX-1 explains the highest anti-inflammatory activity of the compound (Figure 9). The binding of compounds **1** and **7** are shown in Figure 10 and Figure 11, respectively.

## 3. Experimental Section

### 3.1. General Information

All melting points were recorded on open glass capillaries using an Electrothermal IA 9000 digital melting point apparatus and are uncorrected. Analytical data were obtained from the Microanalytical Unit, Cairo University (Egypt). IR spectra (KBr discs) were recorded on a FT-IR Perkin Elmer Spectrum BX spectrophotometer and reported in cm^−1^. The nuclear magnetic resonance spectra were recorded using a Jeol spectrometer (^1^H-NMR: 400 MHz; ^13^C-NMR: 100 MHz) in CD_3_OD and the chemical shifts (δ) were recorded in (ppm) relative to TMS. The mass spectra (MS) were recorded at 70 eV using a Shimadzu QP1000 EX GCMS, using the Electron Ionization (EI) technique. Monitoring of the reactions and checking the purity of the compounds were done by TLC on silica gel pre-coated aluminum sheets (Type 60F_254_, Merck, Darmstadt, Germany) and the spots were detected by exposure to a UV lamp at λ_254_ nanometers for few seconds.

#### 3.1.1. 7-Hydroxy-4-methyl-2*H*-chromen-2-one (**III**)

A solution of resorcinol (**I**, 11 g, 100 mmol) in ethyl acetoacetate (**II**, 8.81 g, 100 mmol) was added dropwise (about 2 h) to stirring H_2_SO_4_ kept at 0 °C. After completion of the addition, the reaction mixture was kept stirring for 24 h at room temperature and then poured onto ice-water. The precipitate formed was collected, washed with cold water and dissolved in 100 mL of 5% aqueous sodium hydroxide solution. The alkaline solution was then acidified with dilute (1:10) sulfuric acid. The separated product was collected, washed several times with cold water and crystallized from ethyl alcohol. Yield 90%, m.p. 185–186 °C (as reported) [16].

#### 3.1.2. 7-Hydroxy-4-methyl-6-nitro-2*H*-chromen-2-one (**IV**)

To a solution of **III** (17.6 g; 100 mmoL) in sulfuric acid (30 mL) kept at a temperature below 5 °C, a mixture of nitric acid (8 mL) and sulfuric acid (10 mL), was added dropwise with stirring so as to keep the temperature below 5 °C. After complete addition (about one hour), the reaction mixture was stirred for another hour at the same temperature and then poured onto ice-water. The yellow precipitate obtained was filtered off, washed with water several times and air-dried. The 6-nitro isomer was obtained using boiling ethanol to separate the freely soluble 8-nitro isomer. Yield 25%, m.p. 262 °C (as reported) [17].

#### 3.1.3. 6-Amino-7-hydroxy-4-methyl-2*H*-chromen-2-one (**V**)

A suspension of **IV** (22.1 g, 100 mmol) and stannous chloride dihydrate (100 g) in ethyl alcohol (100 mL) and HCl (100 mL) was boiled to give a clear solution. The separated solid obtained after cooling in the refrigerator for 2 days was filtered, suspended in water and neutralized with sodium bicarbonate. The resulted yellow precipitate was filtered, extracted several times with hot isopropyl alcohol and the extracts were collected, concentrated under reduced pressure and cooled. The separated products were filtered and crystallized from isopropyl alcohol. Yield 70%, m.p. 273–274 °C (as reported) [18].

*6-(Substituted benzylamino)-7-hydroxy-4-methyl-2H-chromen-2-ones* **1**–**11**. Compound **V** (191 mg, 1.0 mmol), an aromatic aldehyde (1.2 mmol) and acetic acid (100 μL) were dissolved in methanol (5 mL) and stirred at room temperature for 30 min. NaBH_3_CN (126 mg, 2.0 mmol) was added and the reaction mixture was stirred at room temperature for 1 h. Methanol was evaporated and the reaction mixture was extracted with chloroform. The organic layer was washed with brine, dried over anhydrous MgSO_4_, filtered and evaporated. The residue was purified with silica gel column chromatography (chloroform-methanol = 20:1) to give compounds **1**–**11**.

*6-(4-Hydroxybenzylamino)-7-hydroxy-4-methyl-2H-chromen-2-one* (**1**). Yield 83%, m.p. 212–213 °C. IR (KBr disk) υ (cm^−1^); 3480, 3413, 3235, 1684. MS (relative intensity) *m*/*z*; 297 (M^+^, 12), 242 (12), 191 (100), 163 (54), 107 (23). ^1^H-NMR (CD_3_OD) δ 2.30 (s, 3H), 4.30 (s, 2H), 6.04 (s, 1H), 6.65 (s, 1H), 6.68 (s, 1H), 6.76 (d, *J* = 8.6 Hz, 2H), 7.23 (d, *J* = 8.7 Hz, 2H). Anal. calcd. for C_17_H_15_NO_4_ (297.31): C, 68.68; H, 5.09; N, 4.71. Found: C, 68.35; H, 4.93; N, 5.05.

*6-(4-Nitrobenzylamino)-7-hydroxy-4-methyl-2H-chromen-2-one* (**2**). Yield 87%, m.p. 185–186 °C. IR (KBr disk) υ (cm^−1^); 3546, 3400, 1670, 1512, 1347. MS (relative intensity) *m*/*z*; 326 (M^+^, 4), 294 (5), 191 (7), 162 (5), 98 (100). ^1^H-NMR (CD_3_OD): δ 2.22 (s, 3H), 3.35 (s, 2H), 6.02 (s, 1H), 6.50 (s, 1H), 6.71 (s, 1H), 7.65 (d, *J* = 8.8 Hz, 2H), 8.21 (d, *J* = 8.8 Hz, 2H). ^13^C-NMR (CD_3_OD): δ 18.97, 47.91, 102.12, 104.3, 110.84, 113.65, 124.66, 129.11, 136.33, 148.49, 148.53, 149.5, 151.17, 156.12, 164.67. Anal. calcd. for C_17_H_14_N_2_O_5_ (326.31): C, 62.58; H, 4.32; N, 8.58. Found: C, 62.53; H, 4.29; N, 8.61.

*6-(4-(Dimethylamino)benzylamino)-7-hydroxy-4-methyl-2H-chromen-2-one* (**3**). Yield 91%, m.p. 205–216 °C. IR (KBr disk) υ (cm^−1^); 3546, 3392, 1684, 1290. MS (relative intensity) *m*/*z*; 324 (M^+^, 21), 309 (26), 191 (100), 162 (42), 107 (40). Anal. calcd. for C_19_H_20_N_2_O_3_ (324.38): C, 70.35; H, 6.21; N, 8.64. Found: C, 70.39; H, 6.22; N, 8.66.

*N-(4-((7-Hydroxy-4-methyl-2-oxo-2H-chromen-6-ylamino)methyl)phenyl)acetamide* (**4**). Yield 88%, m.p. 235–236 °C. IR (KBr disk) υ (cm^−1^); 3481, 3413, 1675, 1654. MS (relative intensity) *m*/*z*; 336 (M^+^ − 2, 81), 294 (25), 191 (33), 163 (22), 106 (100). ^1^H-NMR (CD_3_OD): δ 2.11 (s, 3H), 2.27 (s, 3H), 4.39 (s, 2H), 6.03 (s, 1H), 6.61 (s, 1H), 6.69 (s, 1H), 7.36 (d, *J* = 8.5 Hz, 2H), 7.50 (d, *J* = 8.5 Hz, 2H). ^13^C-NMR (CD_3_OD): δ 18.78, 23.75, 49.21, 101.95, 104.57, 110.7, 113.7, 121.41, 128.83, 130.51, 135.11, 136.67, 148.38, 151.14, 156.31, 164.79, 171.64. Anal. calcd. for C_19_H_18_N_2_O_4_ (338.37): C, 67.45; H, 5.36; N, 8.28. Found: C, 67.42; H, 5.38; N, 8.31.

*6-(4-Bromobenzylamino)-7-hydroxy-4-methyl-2H-chromen-2-one* (**5**). Yield 89%, m.p. 251–252 °C. IR (KBr disk) υ (cm^−1^); 3481, 3410, 1670. MS (relative intensity) *m*/*z*; 361 (M^+^ + 2, 15), 359 (M^+^, 16), 190 (38), 171 (85), 169 (88), 90 (100). ^1^H-NMR (CD_3_OD): δ 2.25 (s, 3H), 4.41 (s, 2H), 6.03 (s, 1H), 6.54 (s, 1H), 6.69 (s, 1H), 7.33 (d, *J* = 8.3 Hz, 2H), 7.47 (d, *J* = 8.3 Hz, 2H). ^13^C-NMR (CD_3_OD): δ 18.82, 64.39, 102.0, 104.42, 110.78, 113.68, 121.56, 130.24, 132.61, 136.56, 140.48, 148.41, 151.07, 156.2, 164.73. Anal. calcd. for C_17_H_14_BrNO_3_ (360.21): C, 56.69; H, 3.92; N, 3.89. Found: C, 56.71; H, 3.95; N, 3.85.

*6-(5-Bromo-2-hydroxybenzylamino)-7-hydroxy-4-methyl-2H-chromen-2-one* (**6**). Yield 87%, m.p. 243–245 °C. IR (KBr disk) υ (cm^−1^); 3476, 3411, 1699. MS (relative intensity) *m*/*z*; 377 (M^+^ + 2, 11), 375 (M^+^, 12), 191 (100), 163 (51), 162 (35). ^1^H-NMR (CD_3_OD): δ 2.32 (s, 3H), 4.38 (s, 2H), 6.04 (s, 1H), 6.71 (m, 3H), 7.15 (dd, *J* = 2.6, 8.8 Hz, 1H), 7.39 (d, *J* = 2.5 Hz, 1H); ^13^C-NMR (CD_3_OD): δ 18.93, 43.22, 102.02, 104.92, 110.67, 112.29, 113.69, 117.71, 129.28, 131.71, 132.61, 136.52, 148.59, 151.48, 155.93, 156.36, 164.83. Anal. calcd. for C_17_H_14_BrNO_4_ (376.21): C, 54.28; H, 3.75; N, 3.72. Found: C, 54.25; H, 3.74; N, 3.75.

*6-(5-Bromo-2-methoxybenzylamino)-7-hydroxy-4-methyl-2H-chromen-2-one* (**7**). Yield 93%, m.p. 218–219 °C. IR (KBr disk) υ (cm^−1^); 3546, 3412, 1676. MS (relative intensity) *m/z*; 391 (M^+^ + 2, 29), 389 (M^+^, 30), 201 (94), 199 (100), 191 (41), 162 (31). ^1^H-NMR (CD_3_OD): δ 2.30 (s, 3H), 3.89 (s, 3H), 4.39 (s, 2H), 6.04 (s, 1H), 6.62 (s, 1H), 6.69 (s, 1H), 6.92 (d, *J* = 8.5 Hz, 1H), 7.33 (d, *J* = 7.3 Hz, 1H), 7.43 (s, 1H); ^13^C-NMR (CD_3_OD): δ 18.89, 43.09, 56.27, 102.08, 104.57, 110.80, 113.43, 113.78, 122.56, 131.03, 131.94, 132.34, 136.51, 148.50, 151.25, 156.20, 158.07, 164.77. Anal. calcd. for C_18_H_16_BrNO_4_ (390.24): C, 55.40; H, 4.13; N, 3.59. Found: C, 55.43; H, 4.15; N, 3.55.

*6-(3-Hydroxy-4-methoxybenzylamino)-7-hydroxy-4-methyl-2H-chromen-2-one* (**8**). Yield 79%, m.p. 188–189 °C. IR (KBr disk) υ (cm^−1^); 3546, 3414, 1654. MS (relative intensity) *m*/*z*; 327 (M^+^, 24), 191 (35), 162 (18), 137 (100). Anal. calcd. for C_18_H_17_NO_5_ (327.34): C, 66.05; H, 5.23; N, 4.28. Found: C, 66.09; H, 5.19; N, 4.31.

*6-(3-Nitrobenzylamino)-7-hydroxy-4-methyl-2H-chromen-2-one* (**9**). Yield 82%, m.p. 233–235 °C. IR (KBr disk) υ (cm^−1^); 3546, 3400, 1670, 1512, 1347. MS (relative intensity) *m*/*z*; 326 (M^+^, 9), 309 (29), 271 (31), 148 (100), 120, (59). ^1^H-NMR (CD_3_OD): δ 2.36 (s, 3H), 3.35 (s, 2H), 6.02 (s, 1H), 6.55 (s, 1H), 6.72 (s, 1H), 7.58 (t, *J* = 8.0 Hz, 1H), 7.83 (d, *J* = 77.1 Hz, 1H), 8.11 (d, *J* = 8.2 Hz, 1H), 8.3 (s, 1H); ^13^C-NMR (CD_3_OD): δ 18.82, 47.74, 102.19, 104.45, 110.83, 113.66, 122.99, 125,86, 130.76, 133.67, 134.63, 136.25, 143.94, 148.54, 151.18, 156.17, 164.73. Anal. calcd. for C_17_H_14_N_2_O_5_ (326.31): C, 62.58; H, 4.32; N, 8.58. Found: C, 62.55; H, 4.28; N, 8.63.

*6-(4-Hydroxy-3-nitrobenzylamino)-7-hydroxy-4-methyl-2H-chromen-2-one* (**10**). Yield 75%, m.p. 221–223 °C. IR (KBr disc) υ (cm^−1^); 3474, 3420, 1670, 1560, 1330. MS (relative intensity) *m*/*z*; 342 (M^+^, 5), 340 (M^+^ − 2, 100), 202 (33), 191 (59), 163 (32). Anal. calcd. for C_17_H_14_N_2_O_6_ (342.31): C, 59.65; H, 4.12; N, 8.18. Found: C, 59.62; H, 4.15; N, 8.22.

*6-(4-Chloro-3-nitrobenzylamino)-7-hydroxy-4-methyl-2H-chromen-2-one* (**11**). Yield 77%, m.p. 244–245 °C. IR (KBr disc) υ (cm^−1^); 3.473, 3413, 1718, 1533, 1266. MS (relative intensity) *m*/*z*; 363 (M^+^ + 2, 4), 361 (M^+^, 12), 358(51), 202 (59), 191 (57), 163 (52), 80 (100). Anal. calcd. for C_17_H_13_ClN_2_O_5_ (390.24): C, 56.60; H, 3.63; N, 7.77. Found: C, 56.65; H, 3.59; N, 7.81.

### 3.2. Anti-Inflammatory Screening

The anti-inflammatory screening was carried out at the Department of Pharmacology, National Research Center, Dokki, Cairo, Egypt.

#### 3.2.1. Carrageenan-Induced Paw Edema Method

The anti-inflammatory effect of the newly synthesized compounds were evaluated by the carrageenan-induced paw edema method [13]. Male albino Sprague-Dawley rats (175–200 g) were used taking into account the international principles and local regulations concerning the care and the use of laboratory animals [19]. The animal had free access to standard commercial diet and water and were kept at 25 °C room temperature. Thirteen groups of animals each consisting of eight rats were selected. The 1st group was treated with the vehicle and left as control while the 2nd one was given indomethacin by intraperitoneally injection in a dose of 10 mg/kg body weight (reference standard) and tested compounds were injected intraperitoneally at equimolar dose levels. Acute inflammation was induced after 30 min by subplantar injection of 100 µL of 1% suspension of carrageenan (Sigma-Aldrich Co., St. Louis, MO, USA) in the right hind paw of all rats. The paw edema volume (hind foot-pad thickness) was measured at 0 h (immediately after injection of carrageenan), 1 h, 2 h and 3 h using a water Pletysmometer (7141: UGO Basile, Comerio, Italy) [20]. Percent inhibition of the tested compounds and standard drug were calculated in comparison with vehicle control. Carrageenan-induced hind paw edema is the standard experimental model of acute inflammation. Carrageenan is the phlogistic agent of choice for testing anti-inflammatory drugs as it is not known to be antigenic and is devoid of apparent systemic effects. The percentage inhibition was determined for each rat by comparison with the control and values were calculated according to the formula:

% Anti-inflammatory activity = (1 − Rt/Rc) × 100

where Rt is the difference in paw volume of the tested compound group and Rc is the difference in paw volume of the control group.

#### 3.2.2. Statistical Analysis

The anti-inflammatory activity was determined as increase in the paw edema volume percentage in the treated animals (Table 1 and Table 2). Results were expressed as the mean ±SE, and different groups were compared using one way analysis of variance (ANOVA) followed by Tukey-Kramer test for multiple comparisons.

### 3.3. Molecular Docking

Docking simulation study is performed using Molecular Operating Environment (MOE^®^) version 10.2010, Chemical Computing Group Inc. (Montreal, QC, Canada). The computational software operated under Windows XP installed on an Intel Pentium IV PC with a 1.6 GHz processor and 512 MB memory [21].

#### 3.3.1. Target Compounds Optimization

The target compounds were constructed into a 3D model using the builder interface of the MOE program. After checking their structures and formal charges on atoms by 2D depiction. The target compounds were subjected to a conformational search. All conformers were subjected to energy minimization which were performed with MOE until a RMSD gradient of 0.01 Kcal/mole and RMS distance of 0.1 Å with MMFF94X force-field and the partial charges were automatically calculated. The obtained database was then saved as MDB file to be used in the docking calculations.

#### 3.3.2. Optimization of the Enzymes Active Site

The enzymes were prepared for docking studies by the addition of hydrogen atoms to the system with their standard geometry. The atoms connection and type were checked for any errors with automatic correction. Selection of the receptor and its atoms potential were fixed. MOE Alpha Site Finder was used for the active site search in the enzyme structure using all default items. Dummy atoms were created from the obtained alpha Spheres.

#### 3.3.3. Docking of the Target Molecules to the COX-1 and COX-2 Active Sites

Docking of the conformation database of the target compounds was done using MOE-Dock software. The enzyme active site file was loaded and the dock tool was initiated. The program specifications were adjusted to dummy atoms as the docking site, triangle matcher as the placement methodology, London ΔG as scoring methodology and were adjusted to its default values. The MDB file of the ligand to be docked was loaded and dock calculations were run automatically. The obtained poses were studied and the poses that showed best ligand-enzyme interactions were selected and stored for energy calculations.

## 4. Conclusions

In the present study, a series of 6-(substituted benzylamino)-7-hydroxy-4-methyl-*2H*-chromen-2-ones **1**–**11** was synthesized and assessed for their anti-inflammatory activity using the carrageenan-induced hind paw edema method. All the synthesized compounds exhibited significant anti-inflammatory activity, especially compounds **4** and **8** which showed maximum anti-inflammatory activity exceeding that of the reference indomethacin itself. Furthermore, a molecular docking study of all the synthesized compound was carried out to understand the binding interaction between the new compounds with the COX-1 and COX-2 enzymes. The results of this study suggest a good binding interaction which explains the significant biological activity. Further investigation of the binding mode and optimization of the structure of this promising series of compounds will be carried out in the future.

## Supplementary Materials

Supplementary materials can be accessed at: http://www.mdpi.com/1420-3049/20/04/5374/s1.
