# Supplementary material for: Anti-Inflammatory Screening and Molecular Modeling of Some Novel Coumarin Derivatives
Source: Molecules. 2015 Mar 26;20(4):5374–91. doi: 10.3390/molecules20045374 (PMC6272174; doi:10.3390/molecules20045374)
Supplement: Supplementary file 1 [file molecules-20-05374-s001.pdf]

Supplementary Materials

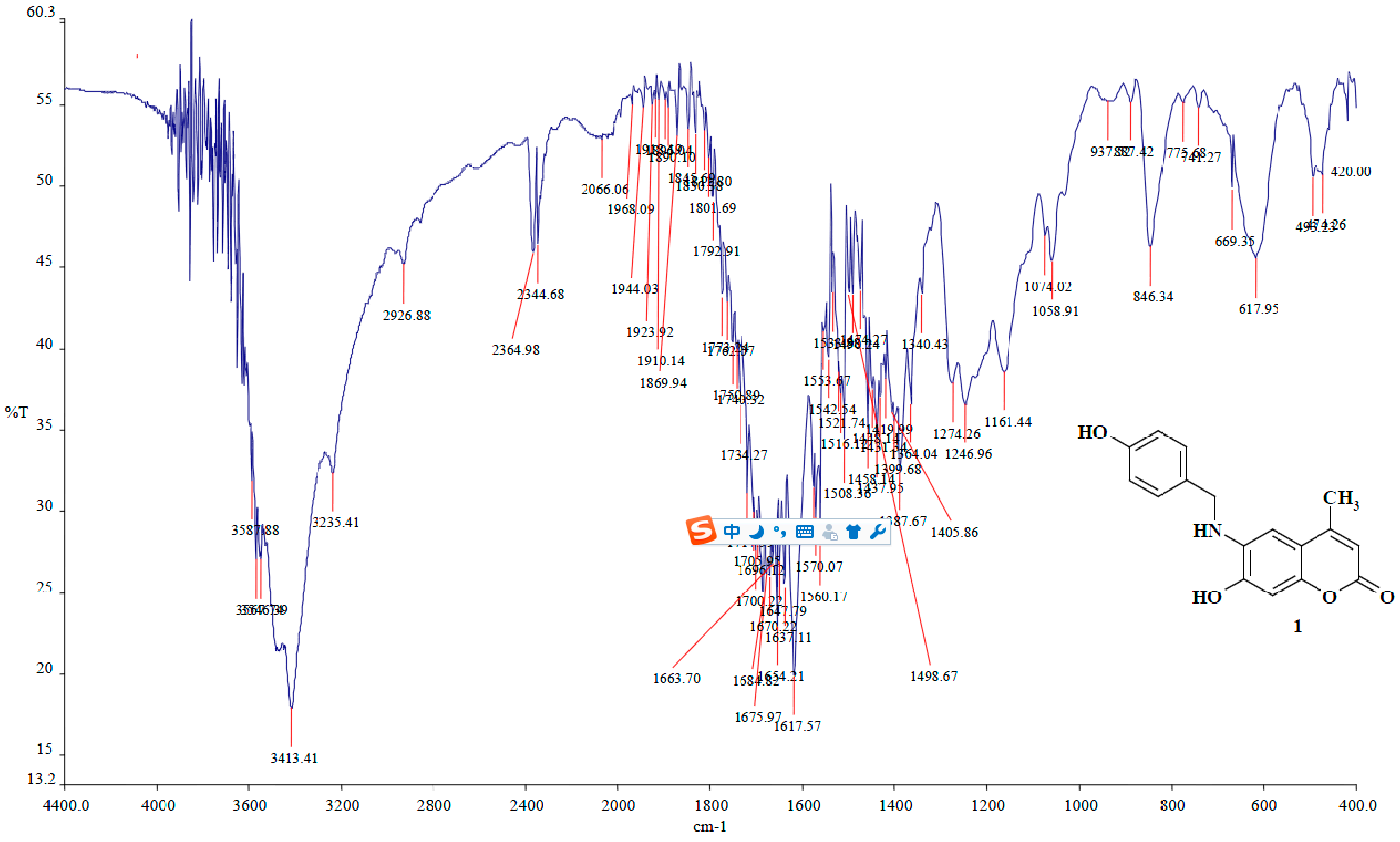

Figure S1. IR of compound **1**.

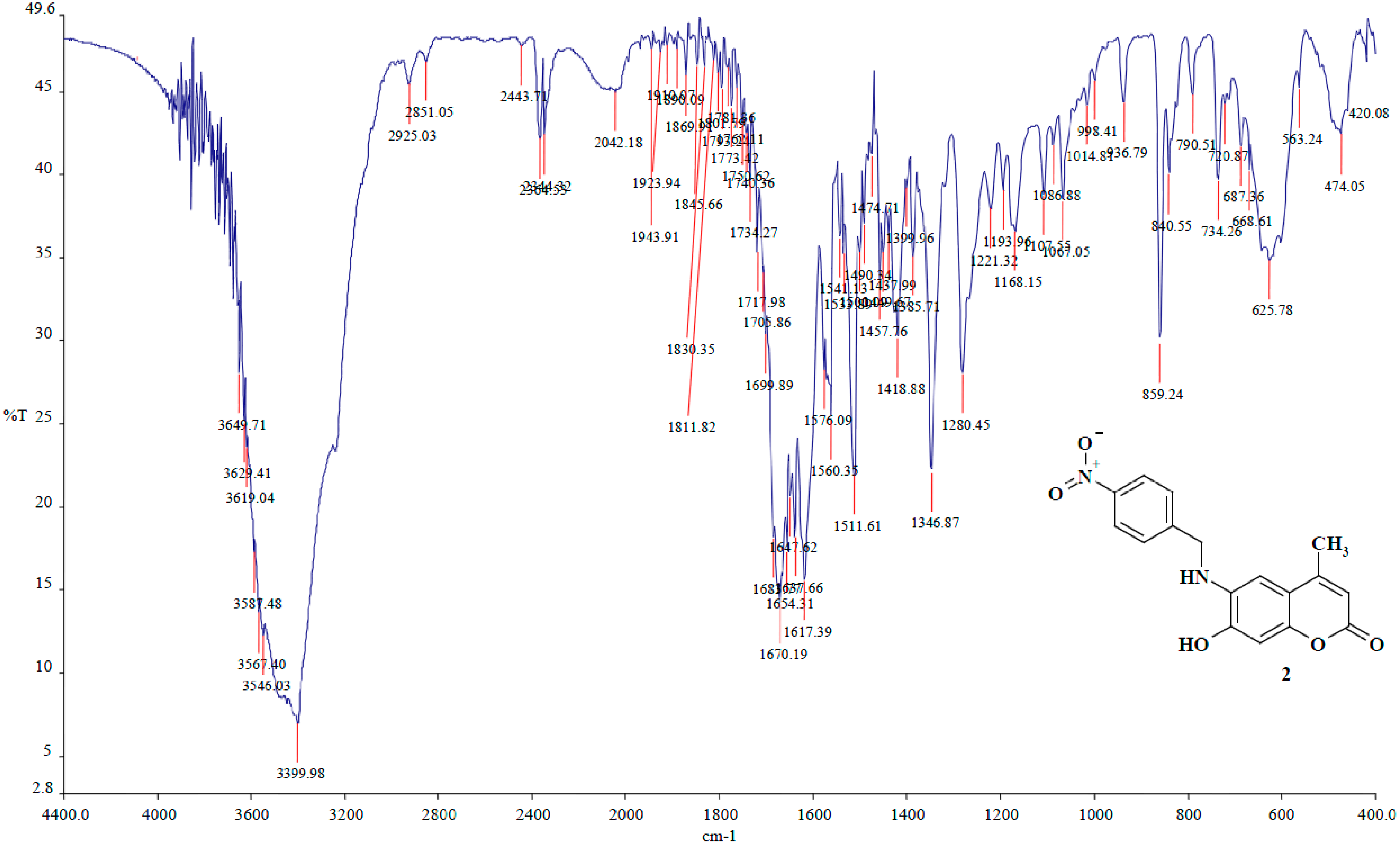

Figure S2. IR of compound **2**.

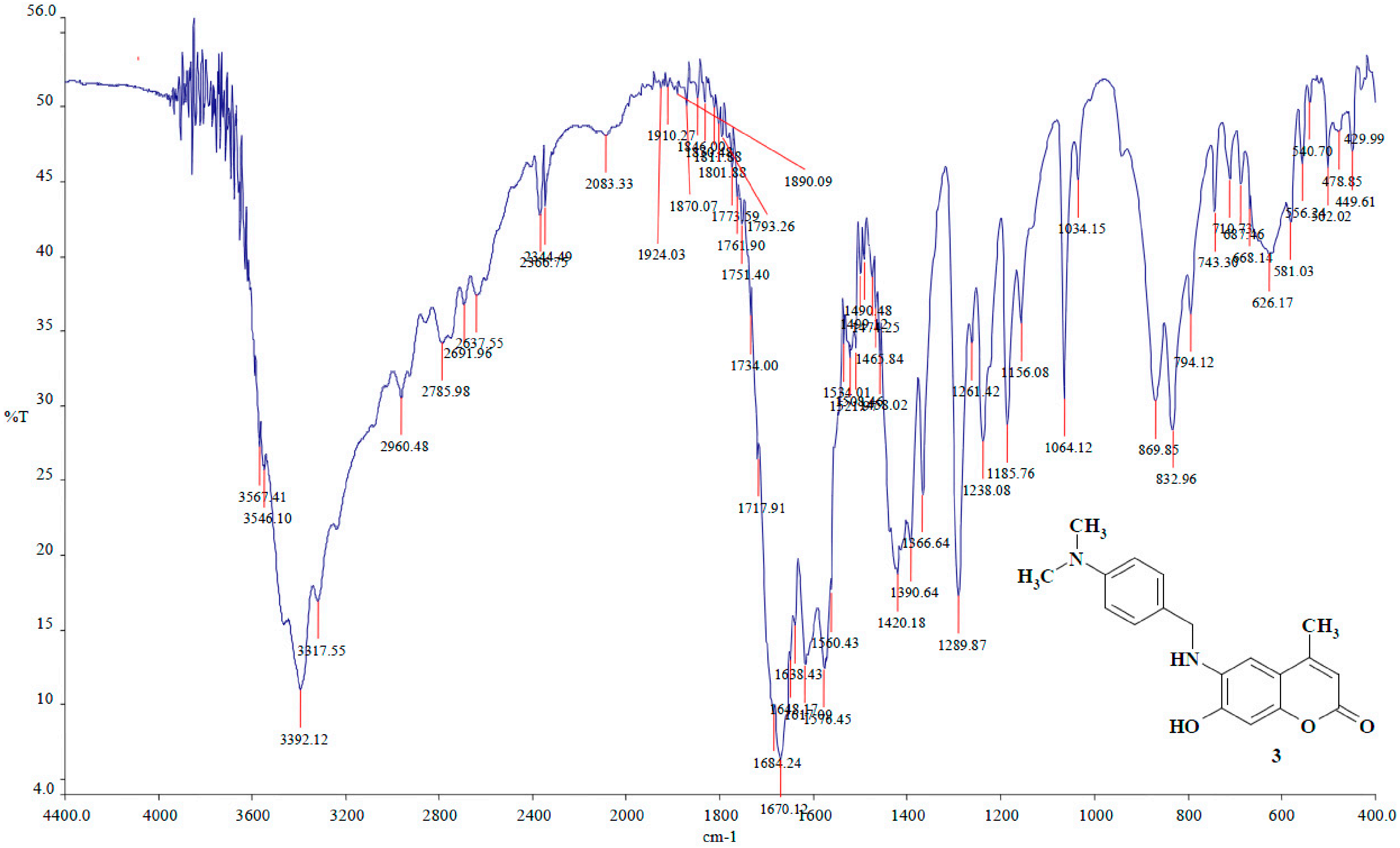

Figure S3. IR of compound **3**.

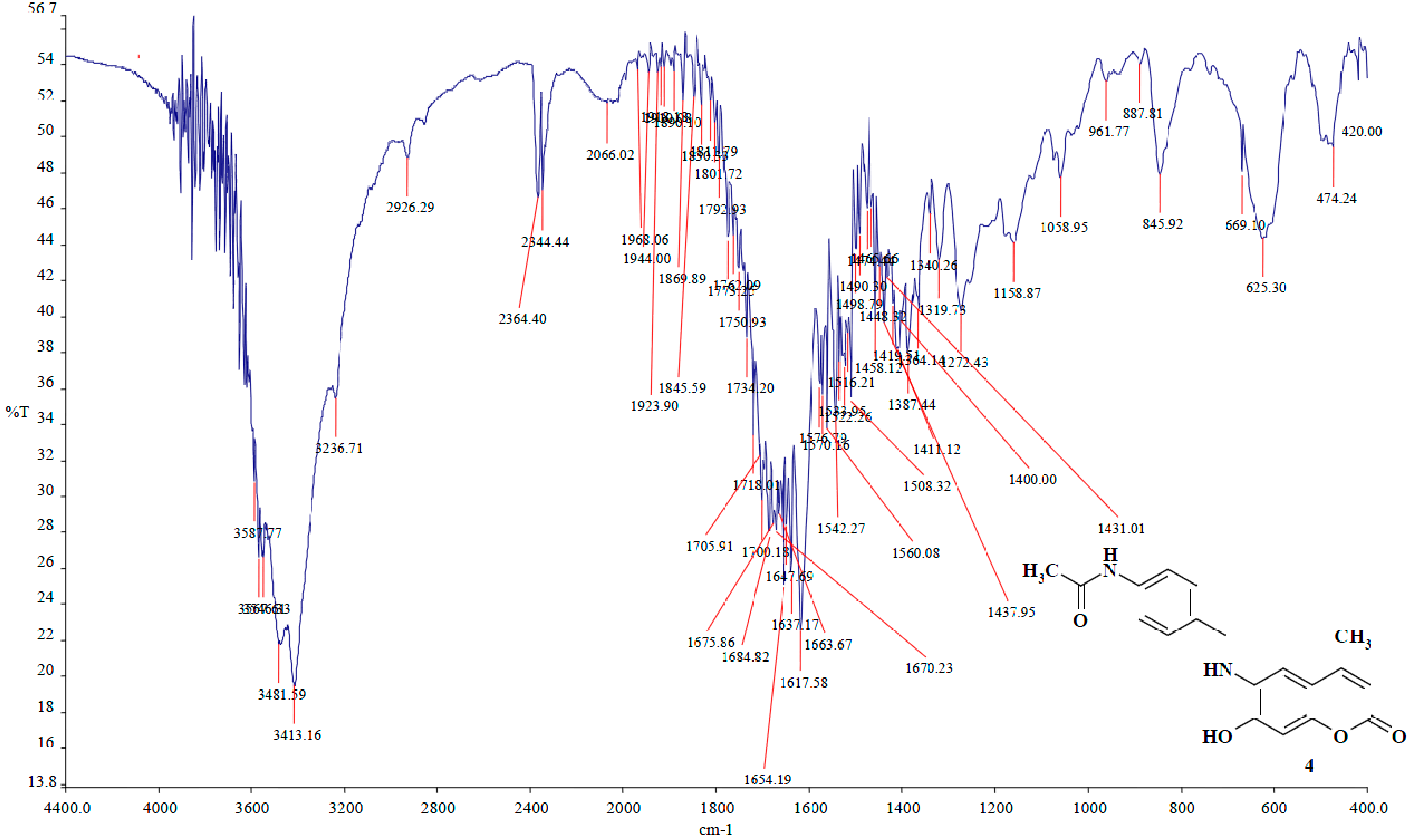

Figure S4. IR of compound **4**.

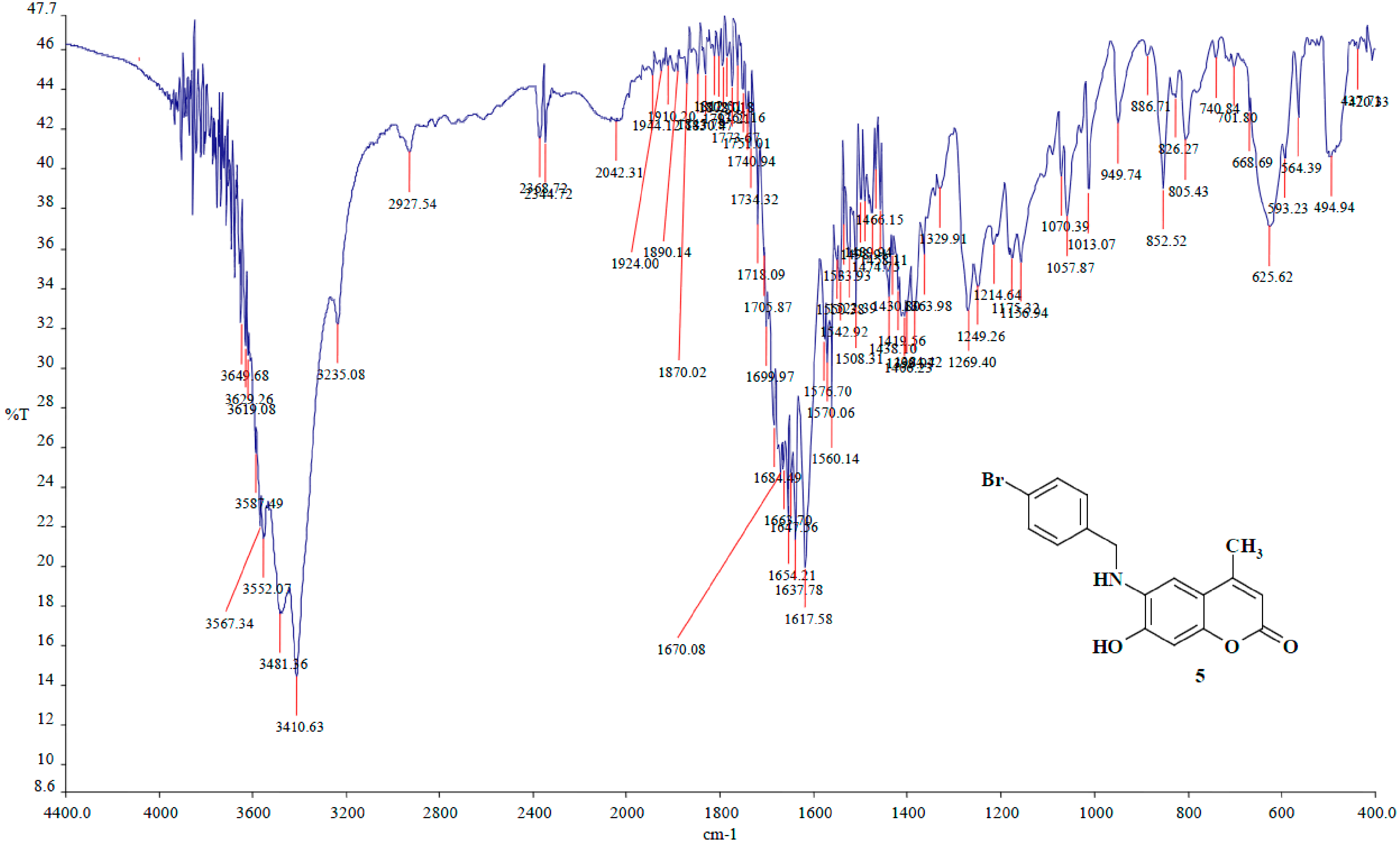

Figure S5. IR of compound **5**.

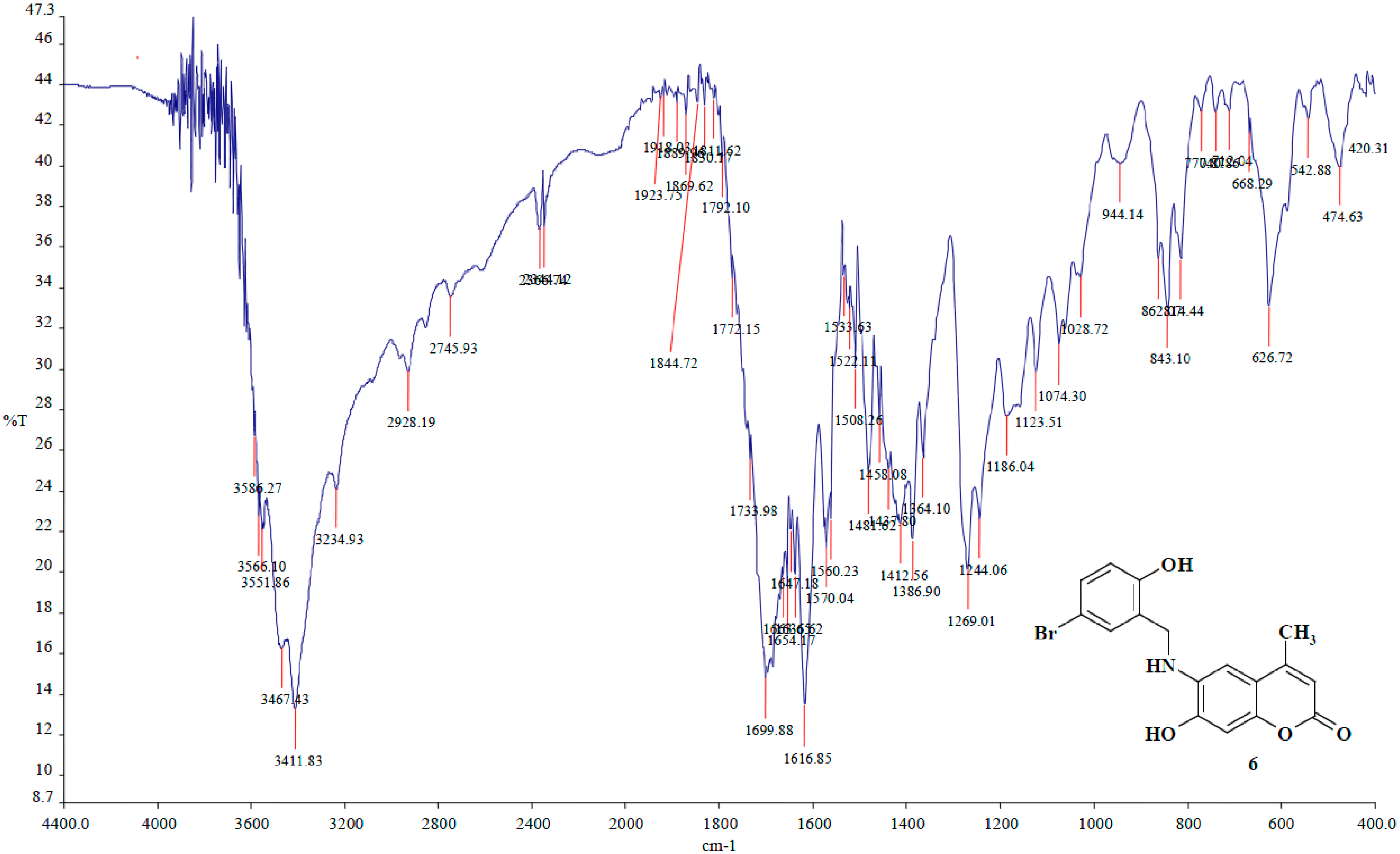

Figure S6. IR of compound **6**.

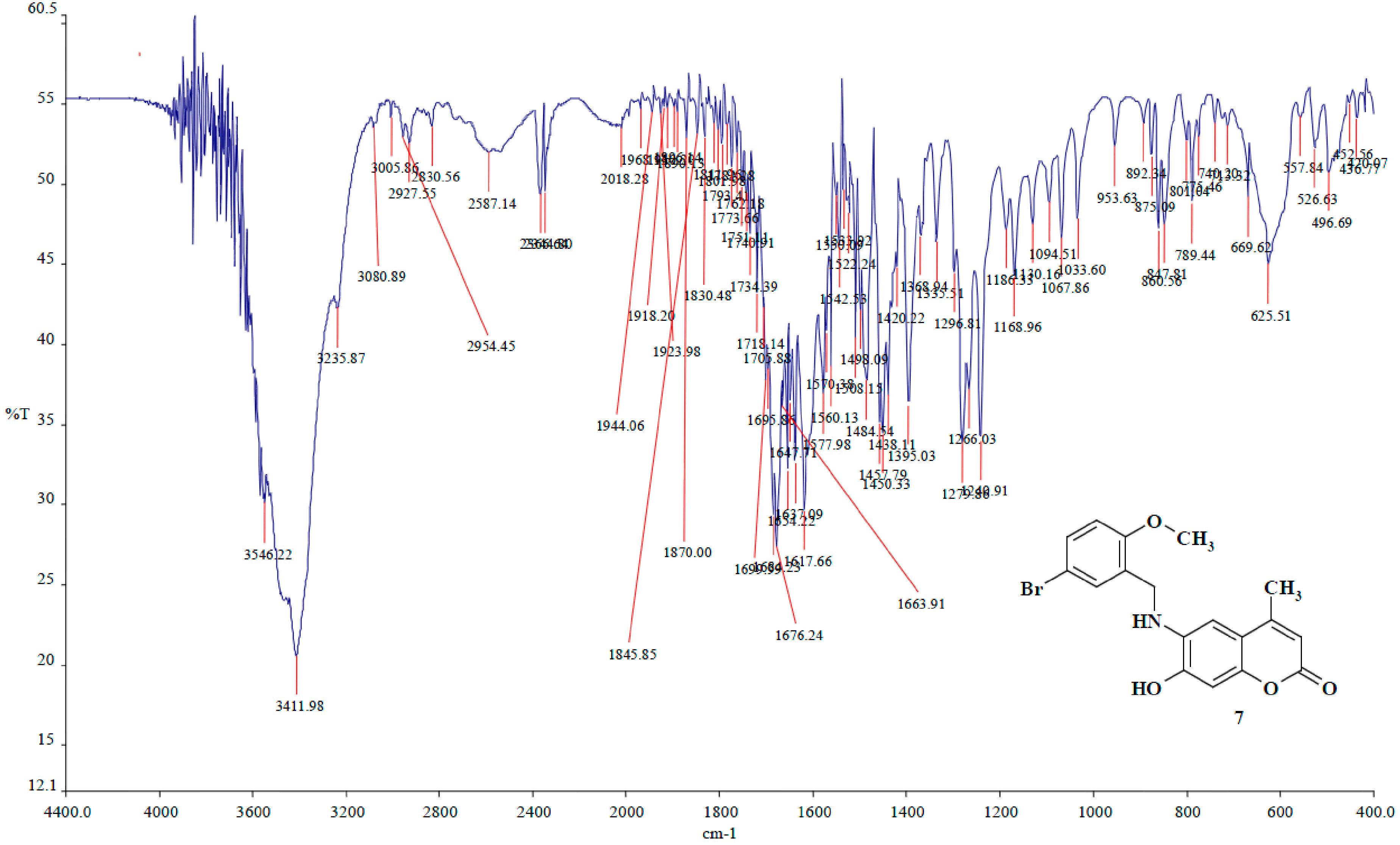

Figure S7. IR of compound **7**.

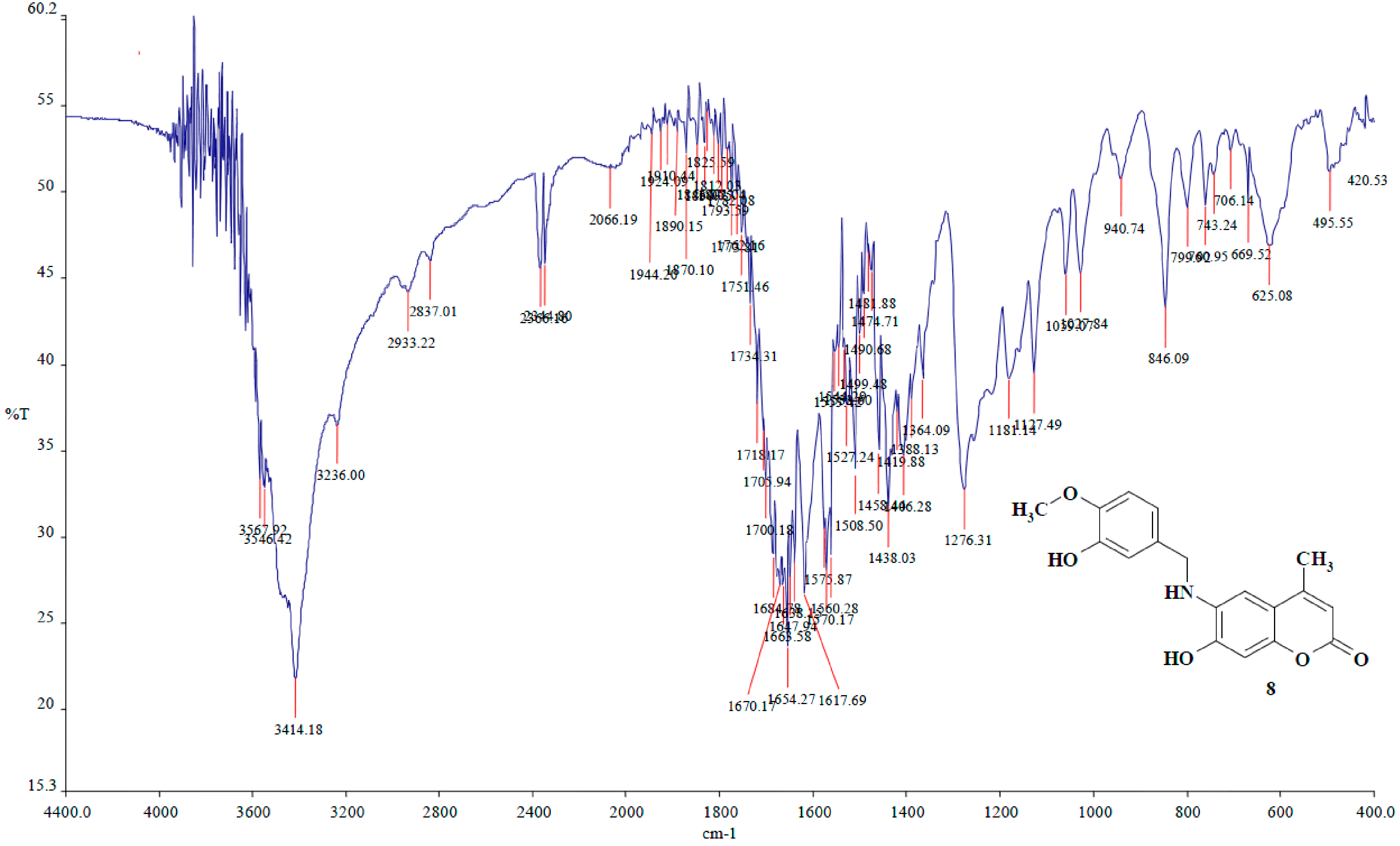

Figure S8. IR of compound **8**.

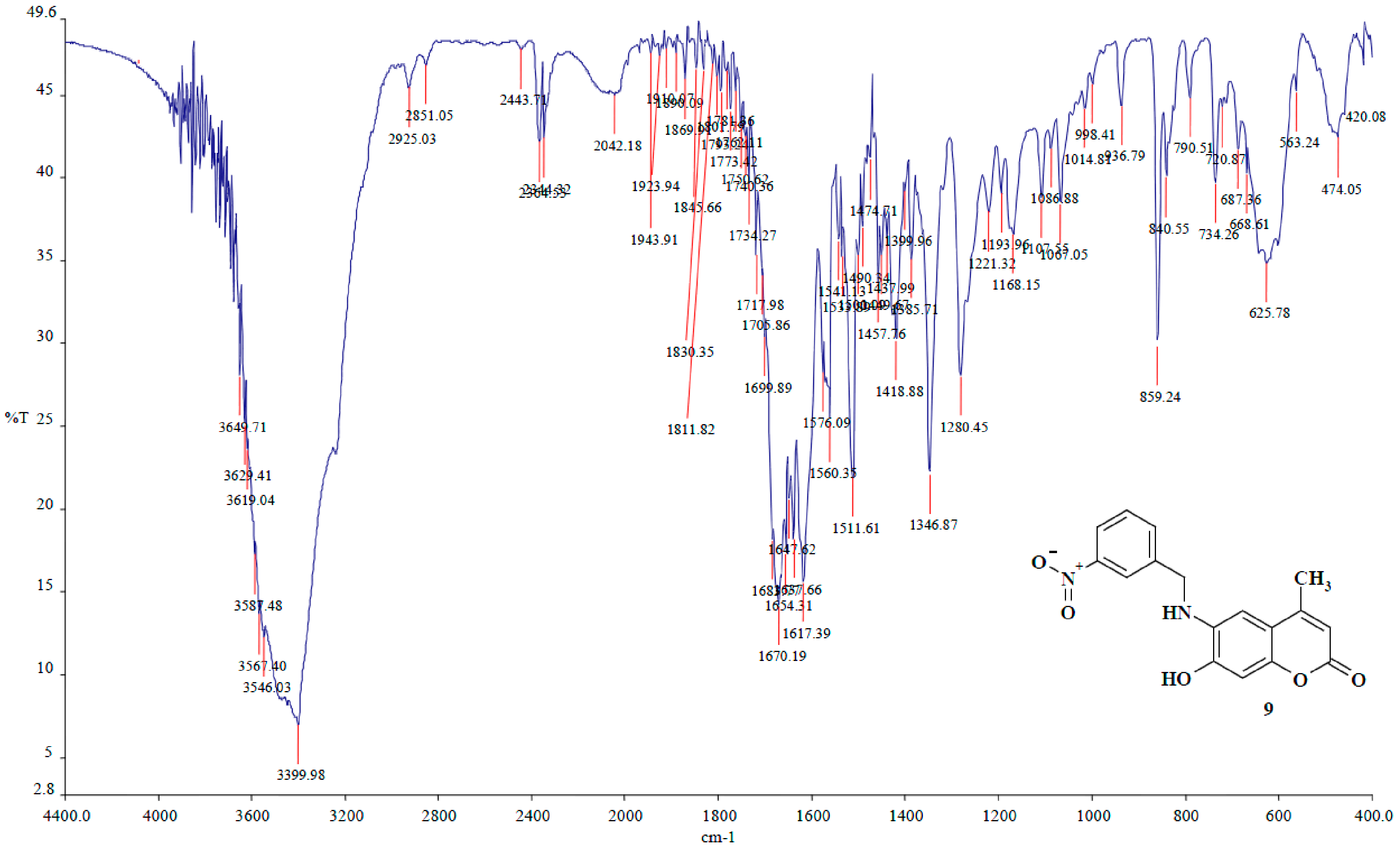

Figure S9. IR of compound **9**.

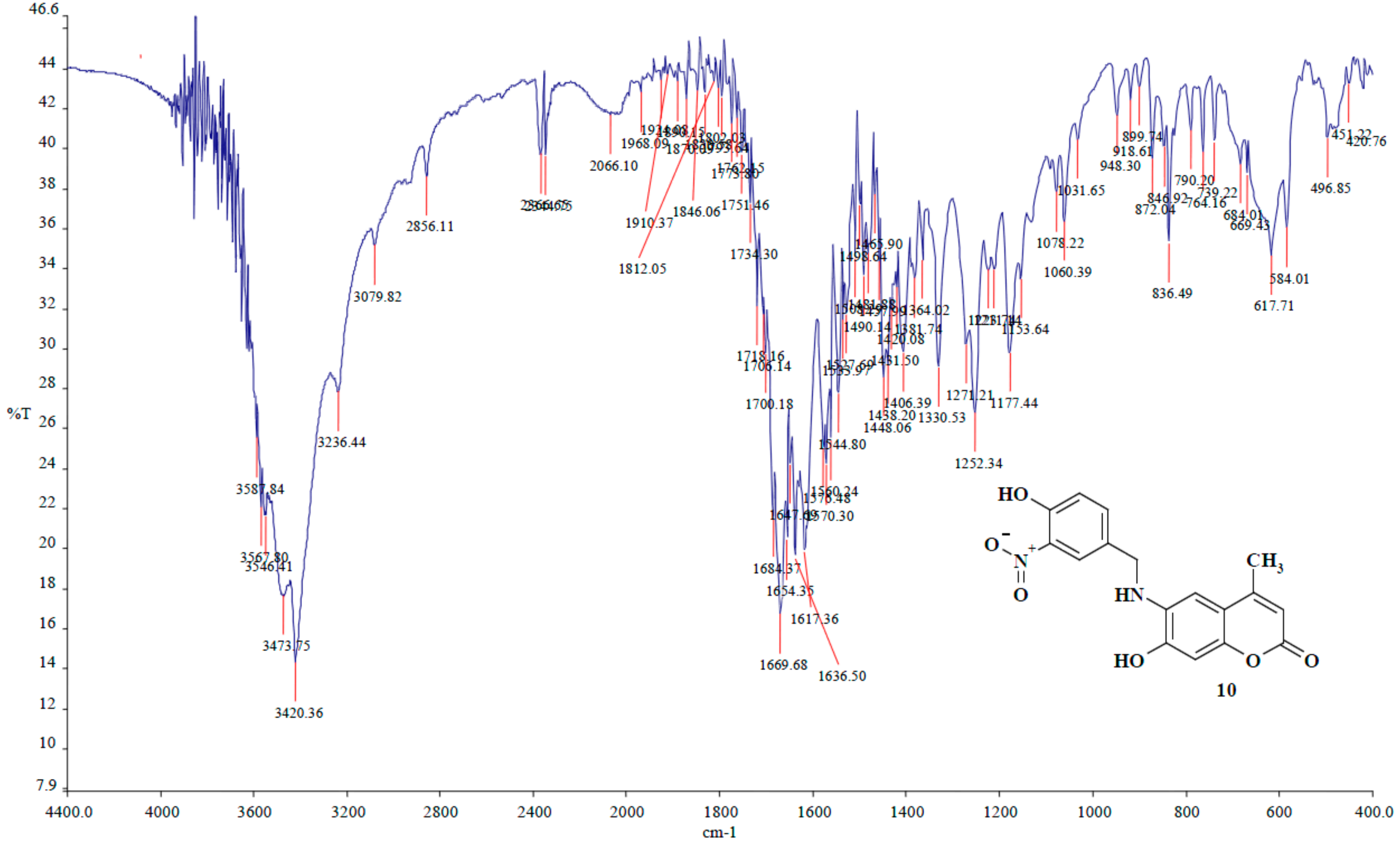

Figure S10. IR of compound **10**.

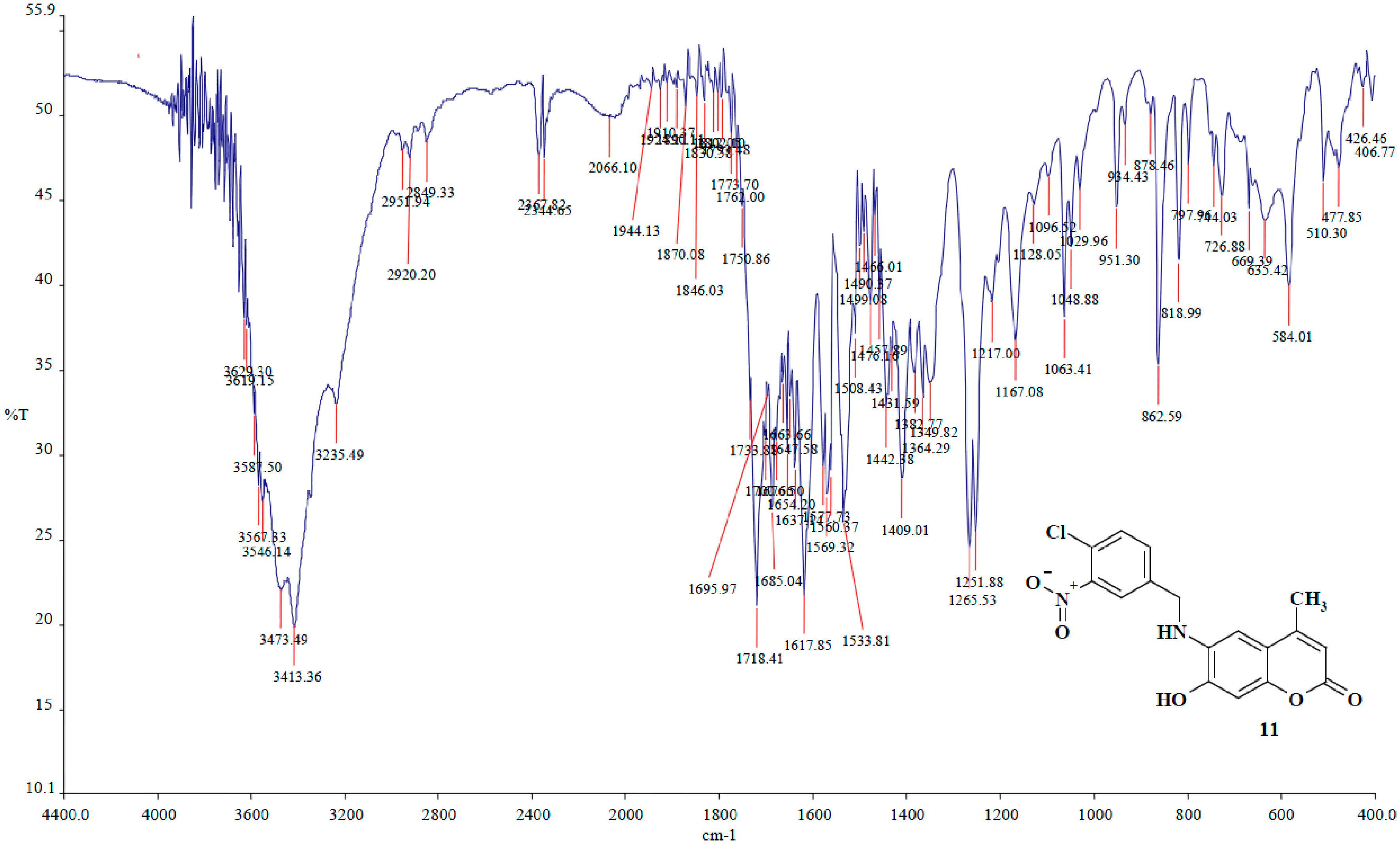

Figure S11. IR of compound **11**.

03-Sep-14 18:10:49

Cairo University
Micro Analytical Center

DI Analysis
Shimadzu Qp-2010 Plus

Sample Information

Analyzed by : Mai Younis
Analyzed : 03/09/2014 06:02:51 م
Sample Name : 6
Sample ID :
Customer Name : Dr.Radwan Saad - Medicine - Helwan
Data File : C:\GCMSsolution\Data\Project1\6.QGD
Org Data File : C:\GCMSsolution\Data\Project1\6.QGD
Method File : C:\GCMSsolution\Data\Project1\A.GABR.qgm
Org Method File : C:\GCMSsolution\Data\Project1\A.GABR.qgm
Report File :
Tuning File : C:\GCMSsolution\System\Tune1_default1.qgt
$EndIf$Modified by : Mai Younis
Modified : 03/09/2014 06:07:26 م

Method

==== Analytical Line 1 ====
IonSourceTemp :250.00 °C
[MS Table]
--Group 1 - Event 1--
Start Time :0.00min
End Time :10.00min
ACQ Mode :Scan
Event Time :0.50sec
Scan Speed :1428
Start m/z :50.00
End m/z :700.00

Electron Voltage : 70 eV
Ionization Mode : EI

C:\GCMSsolution\Data\Project1\6.QGD

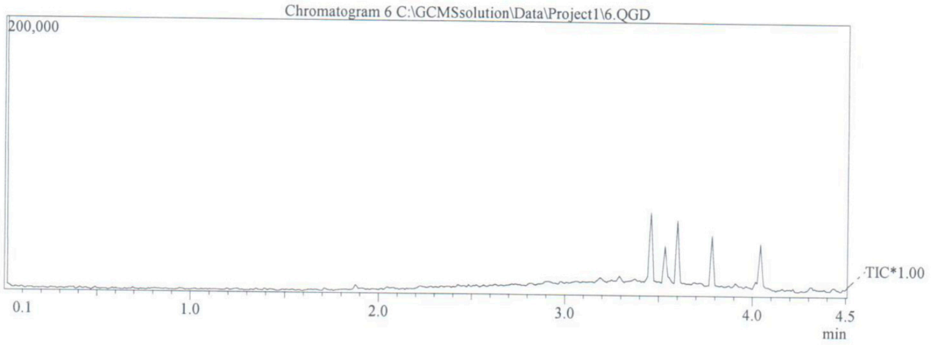

Spectrum

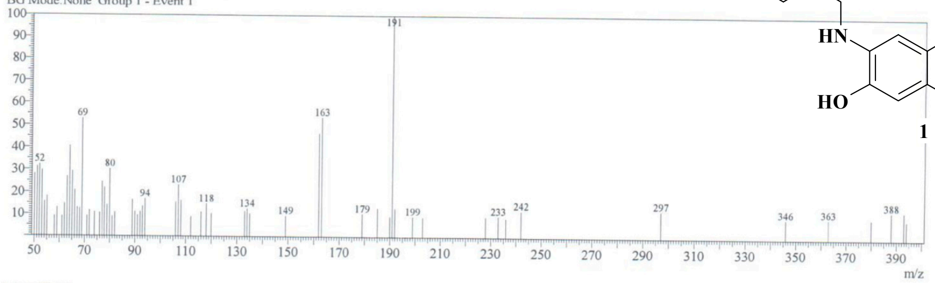

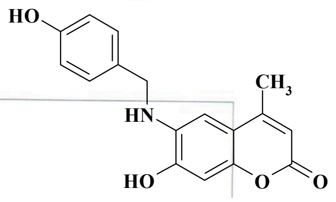

1

Mass Table
Line#:1 R.Time:4.0(Scan#:483)
MassPeaks:64
RawMode:Single 4.0(483) BasePeak:191(937)
BG Mode:None Group 1 - Event 1

#	m/z	Abs. In	Rel. Int.	#	m/z	Abs. In	Rel. Int.	#	m/z	Abs. In	Rel. Int.
1	50.00	263	28.07	4	53.00	278	29.67	7	58.00	86	9.18
2	51.00	294	31.38	5	54.00	146	15.58	8	59.00	122	13.02
3	52.00	303	32.34	6	55.00	170	18.14	9	61.00	84	8.96

Figure S12. Mass Spectra of compound **1**.

03-Sep-14 17:45:42

Cairo University
Micro Analytical Center

DI Analysis
Shimadzu Qp-2010 Plus

Sample Information

Analyzed by	: Mai Younis
Analyzed	: 03/09/2014 05:36:41 م
Sample Name	: 4
Sample ID	:
Customer Name	: Dr.Radwan Saad - Medicine - Helwan
Data File	: C:\GCMSsolution\Data\Project1\4.QGD
Org Data File	: C:\GCMSsolution\Data\Project1\4.QGD
Method File	: C:\GCMSsolution\Data\Project1\A.GABR.qgm
Org Method File	: C:\GCMSsolution\Data\Project1\A.GABR.qgm
Report File	:
Tuning File	: C:\GCMSsolution\System\Tune1_default1.qgt
$EndIf$Modified by	: Mai Younis
Modified	: 03/09/2014 05:41:10 م

Method

==== Analytical Line 1 ====

IonSourceTemp	:250.00 °C
[MS Table]	
--Group 1 - Event 1--	
Start Time	:0.00min
End Time	:10.00min
ACQ Mode	:Scan
Event Time	:0.50sec
Scan Speed	:1428
Start m/z	:50.00
End m/z	:700.00
Electron Voltage	: 70 eV
Ionization Mode	: EI

C:\GCMSsolution\Data\Project1\4.QGD

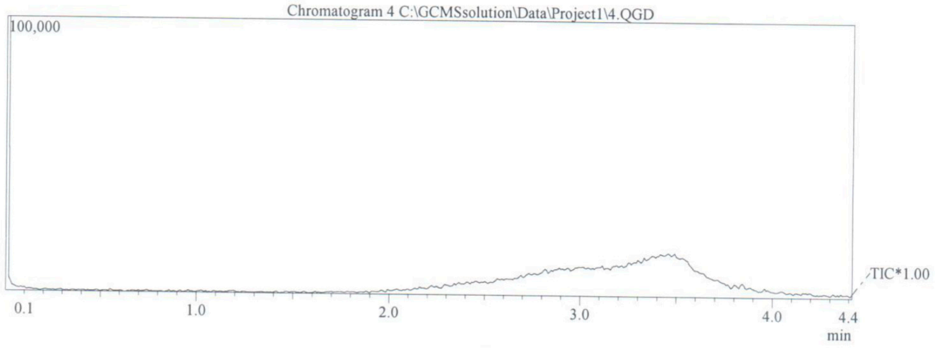

Spectrum

Line#:1 R.Time:3.5(Scan#:416)
MassPeaks:56
RawMode:Single 3.5(416) BasePeak:98(4023)
BG Mode:None Group 1 - Event 1

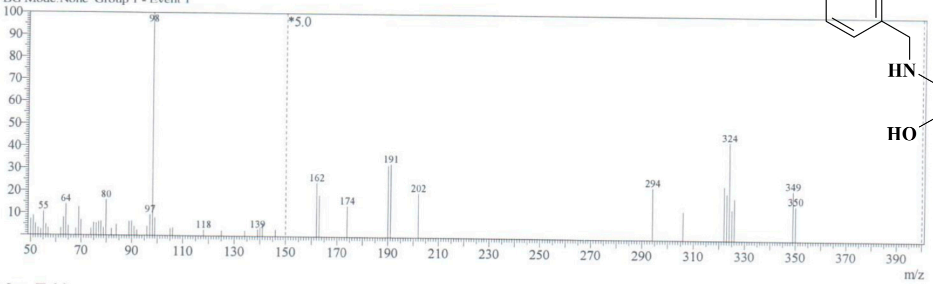

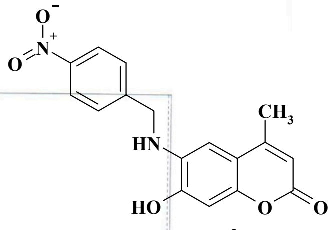

2

Mass Table
Line#:1 R.Time:3.5(Scan#:416)
MassPeaks:56
RawMode: Single 3.5(416) BasePeak:98(4023)
BG Mode:None Group 1 - Event 1

#	m/z	Abs. In	Rel. Int.	#	m/z	Abs. In	Rel. Int.	#	m/z	Abs. In	Rel. Int.
1	50.00	289	7.18	4	53.00	135	3.36	7	56.00	194	4.82
2	51.00	354	8.80	5	54.00	106	2.63	8	57.00	135	3.36
3	52.00	206	5.12	6	55.00	423	10.51	9	62.00	132	3.28

1 / 2

Figure S13. Mass Spectra of compound **2**.

12-Oct-14 20:42:43

Cairo University
Micro Analytical Center

DI Analysis
Shimadzu Qp-2010 Plus

Sample Information

Analyzed by	: A.GABR
Analyzed	: 12/10/2014 08:34:20 م
Sample Name	: 10
Sample ID	:
Customer Name	: رضوان الحجار - صيدله حلوان
Data File	: C:\GCMSsolution\Data\Project1\10.QGD
Org Data File	: C:\GCMSsolution\Data\Project1\10.QGD
Method File	: C:\GCMSsolution\Data\Project1\A.GABR.qgm
Org Method File	: C:\GCMSsolution\Data\Project1\A.GABR.qgm
Report File	:
Tuning File	: C:\GCMSsolution\System\Tune1_default1.qgt
$EndIf$Modified by	: A.GABR
Modified	: 12/10/2014 08:38:47 م

Method

===== Analytical Line 1 =====

IonSourceTemp	:250.00 °C
[MS Table]	
--Group 1 - Event 1--	
Start Time	:0.00min
End Time	:10.00min
ACQ Mode	:Scan
Event Time	:0.50sec
Scan Speed	: 769
Start m/z	:50.00
End m/z	:400.00
Electron Voltage	: 70 eV
Ionization Mode	: EI

C:\GCMSsolution\Data\Project1\10.QGD

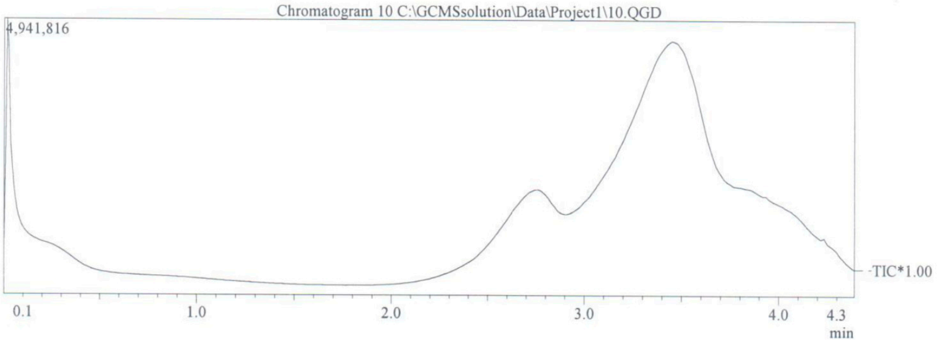

Spectrum

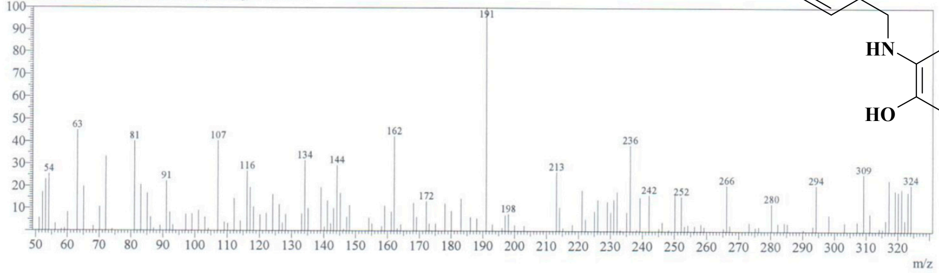

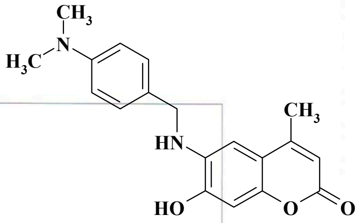

3

Mass Table
Line#:1 R.Time:1.8(Scan#:215)
MassPeaks:136
RawMode:Single 1.8(215) BasePeak:191(339)
BG Mode:Averaged 1.8-1.8(211-211) Group 1 - Event 1

#	m/z	Abs. In	Rel. Int.	#	m/z	Abs. In	Rel. Int.	#	m/z	Abs. In	Rel. Int.
1	51.05	19	5.60	4	54.10	86	25.37	7	59.10	4	1.18
2	52.10	58	17.11	5	56.10	11	3.24	8	60.05	28	8.26
3	53.05	78	23.01	6	58.10	3	0.88	9	63.05	153	45.13

1 / 2

Figure S14. Mass spectra of compound **3**.

03-Sep-14 18:01:53

Cairo University
Micro Analytical Center

DI Analysis
Shimadzu Qp-2010 Plus

Sample Information

Analyzed by	: Mai Younis
Analyzed	: 03/09/2014 05:57:19 م
Sample Name	: 5
Sample ID	:
Customer Name	: Dr.Radwan Saad - Medicine - Helwan
Data File	: C:\GCMSsolution\Data\Project1\ 5.QGD
Org Data File	: C:\GCMSsolution\Data\Project1\ 5.QGD
Method File	: C:\GCMSsolution\Data\Project1\A.GABR.qgm
Org Method File	: C:\GCMSsolution\Data\Project1\A.GABR.qgm
Report File	:
Tuning File	: C:\GCMSsolution\System\Tune1_default1.qgt
$EndIf$Modified by	: Mai Younis
Modified	: 03/09/2014 06:00:54 م

Method

==== Analytical Line 1 ====

IonSourceTemp	:250.00 °C
[MS Table]	
--Group 1 - Event 1--	
Start Time	:0.00min
End Time	:10.00min
ACQ Mode	:Scan
Event Time	:0.50sec
Scan Speed	:1428
Start m/z	:50.00
End m/z	:700.00
Electron Voltage	: 70 eV
Ionization Mode	: EI

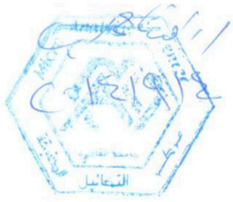

C:\GCMSsolution\Data\Project1\ 5.QGD

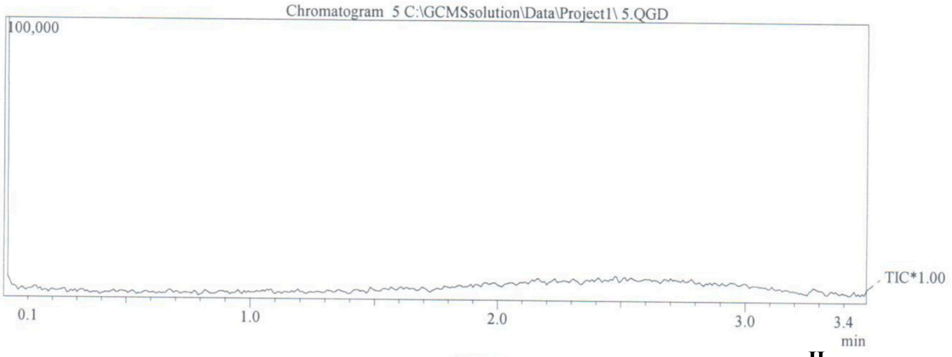

Spectrum

Line#:1 R.Time:2.8(Scan#:334)
MassPeaks:48
RawMode:Single 2.8(334) BasePeak:106(567)
BG Mode:None Group 1 - Event 1

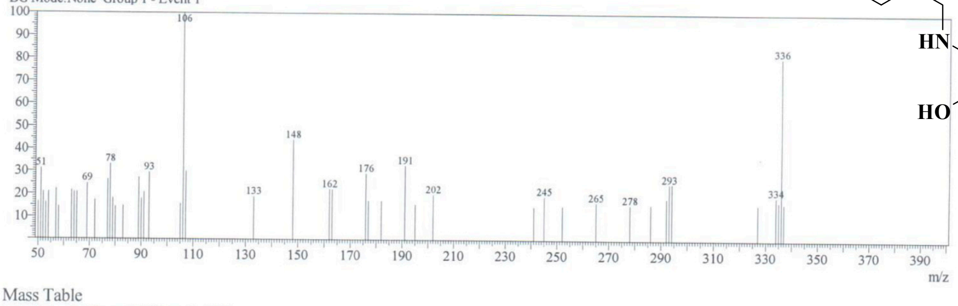

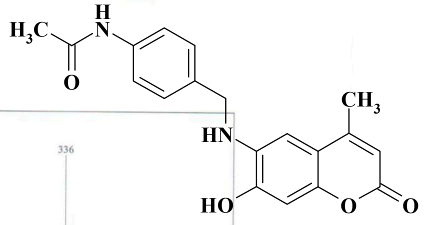

4

Mass Table
Line#:1 R.Time:2.8(Scan#:334)
MassPeaks:48
RawMode:Single 2.8(334) BasePeak:106(567)
BG Mode:None Group 1 - Event 1

#	m/z	Abs. In	Rel. Int.	#	m/z	Abs. In	Rel. Int.	#	m/z	Abs. In	Rel. Int.
1	50.00	94	16.58	4	53.00	92	16.23	7	58.00	82	14.46
2	51.00	177	31.22	5	54.00	118	20.81	8	63.00	122	21.52
3	52.00	118	20.81	6	57.00	126	22.22	9	64.00	118	20.81

Figure S15. Mass Spectra of compound **4**.

03-Sep-14 17:42:34

Cairo University
Micro Analytical Center

DI Analysis
Shimadzu Qp-2010 Plus

Sample Information

Analyzed by	: Mai Younis
Analyzed	: 03/09/2014 05:24:53 م
Sample Name	: 2
Sample ID	:
Customer Name	: Dr.Radwan Saad - Medicine - Helwan
Data File	: C:\GCMSsolution\Data\Project1\2.QGD
Org Data File	: C:\GCMSsolution\Data\Project1\2.QGD
Method File	: C:\GCMSsolution\Data\Project1\A.GABR.qgm
Org Method File	: C:\GCMSsolution\Data\Project1\A.GABR.qgm
Report File	:
Tuning File	: C:\GCMSsolution\System\Tune1_default1.qgt
$EndIf$Modified by	: Mai Younis
Modified	: 03/09/2014 05:28:50 م

Method

==== Analytical Line 1 ====

IonSourceTemp	:250.00 °C
[MS Table]	
--Group 1 - Event 1--	
Start Time	:0.00min
End Time	:10.00min
ACQ Mode	:Scan
Event Time	:0.50sec
Scan Speed	:1428
Start m/z	:50.00
End m/z	:700.00
Electron Voltage	: 70 eV
Ionization Mode	: EI

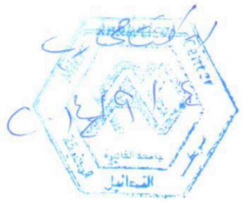

C:\GCMSsolution\Data\Project1\2.QGD

Chromatogram 2 C:\GCMSsolution\Data\Project1\2.QGD

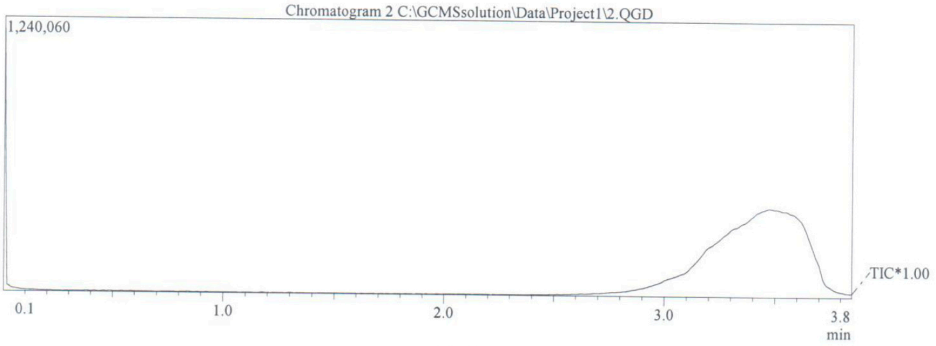

Spectrum

Line#:1 R.Time:3.5(Scan#:418)
MassPeaks:212
RawMode:Single 3.5(418) BasePeak:90(29231)
BG Mode:None Group 1 - Event 1

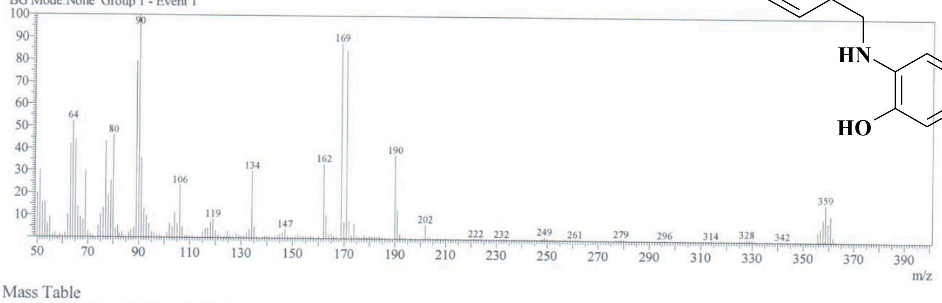

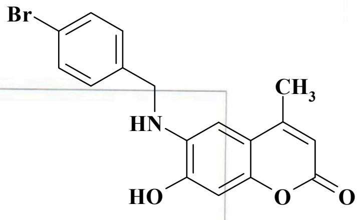

5

Mass Table
Line#:1 R.Time:3.5(Scan#:418)
MassPeaks:212
RawMode:Single 3.5(418) BasePeak:90(29231)
BG Mode:None Group 1 - Event 1

#	m/z	Abs. In	Rel. Int.	#	m/z	Abs. In	Rel. Int.	#	m/z	Abs. In	Rel. Int.
1	49.95	5753	19.68	4	52.95	4649	15.90	7	56.00	412	1.41
2	50.95	8804	30.12	5	53.95	1878	6.42	8	57.00	650	2.22
3	51.95	4586	15.69	6	54.95	2699	9.23	9	57.80	151	0.52

1 / 2

Figure S16. Mass Spectra of compound **5**.

03-Sep-14 17:40:57

Cairo University
Micro Analytical Center

DI Analysis
Shimadzu Qp-2010 Plus

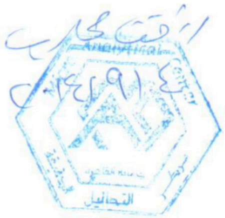

Sample Information

Analyzed by	: Mai Younis
Analyzed	: 03/09/2014 05:18:57 م
Sample Name	: 1
Sample ID	:
Customer Name	: Dr.Radwan Saad - Medicine - Helwan
Data File	: C:\GCMSsolution\Data\Project1\1.QGD
Org Data File	: C:\GCMSsolution\Data\Project1\1.QGD
Method File	: C:\GCMSsolution\Data\Project1\A.GABR.qgm
Org Method File	: C:\GCMSsolution\Data\Project1\A.GABR.qgm
Report File	:
Tuning File	: C:\GCMSsolution\System\Tune1_default1.qgt
$EndIf$Modified by	: Mai Younis
Modified	: 03/09/2014 05:23:27 م

Method

==== Analytical Line 1 ====

IonSourceTemp	:250.00 °C
[MS Table]	
--Group 1 - Event 1--	
Start Time	:0.00min
End Time	:10.00min
ACQ Mode	:Scan
Event Time	:0.50sec
Scan Speed	:1428
Start m/z	:50.00
End m/z	:700.00
Electron Voltage	: 70 eV
Ionization Mode	: EI

C:\GCMSsolution\Data\Project1\1.QGD

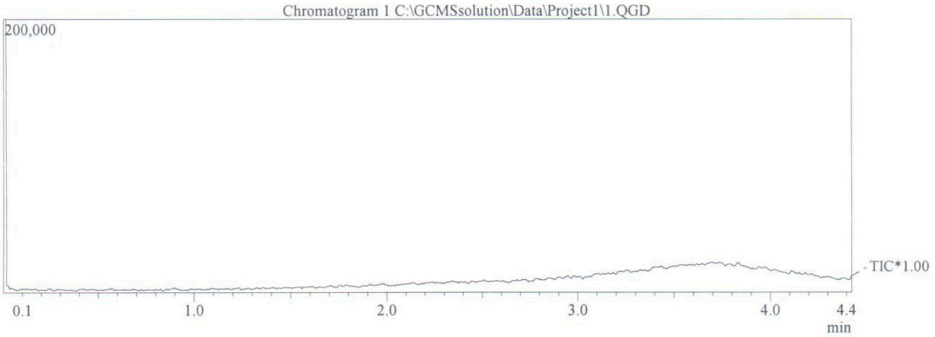

Spectrum

Line#:1 R.Time:3.7(Scan#:440)
MassPeaks:88
RawMode:Single 3.7(440) BasePeak:191(1418)
BG Mode:None Group 1 - Event 1

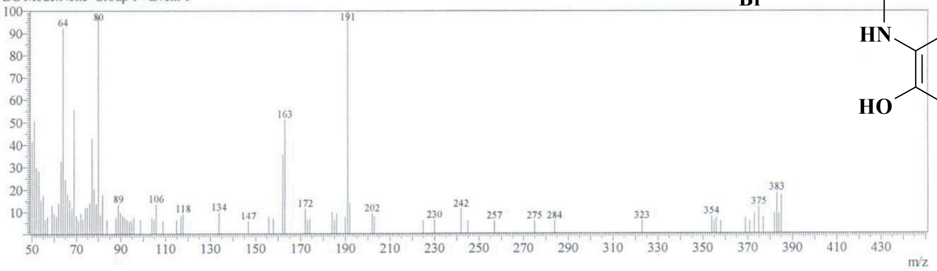

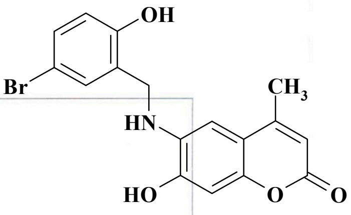

6

Mass Table
Line#:1 R.Time:3.7(Scan#:440)
MassPeaks:88
RawMode:Single 3.7(440) BasePeak:191(1418)
BG Mode:None Group 1 - Event 1

#	m/z	Abs. In	Rel. Int.	#	m/z	Abs. In	Rel. Int.	#	m/z	Abs. In	Rel. Int.
1	49.95	589	41.54	4	52.90	401	28.28	7	55.90	92	6.49
2	50.95	723	50.99	5	53.90	220	15.51	8	56.90	111	7.83
3	51.90	423	29.83	6	54.90	246	17.35	9	58.90	185	13.05

1 / 2

Figure S 17. Mass Spectra of compound **6**.

03-Sep-14 17:44:33

Cairo University
Micro Analytical Center

DI Analysis
Shimadzu Qp-2010 Plus

Sample Information

Analyzed by	: Mai Younis
Analyzed	: 03/09/2014 05:30:49 م
Sample Name	: 3
Sample ID	:
Customer Name	: Dr.Radwan Saad - Medicine - Helwan
Data File	: C:\GCMSsolution\Data\Project1\3.QGD
Org Data File	: C:\GCMSsolution\Data\Project1\3.QGD
Method File	: C:\GCMSsolution\Data\Project1\A.GABR.qgm
Org Method File	: C:\GCMSsolution\Data\Project1\A.GABR.qgm
Report File	:
Tuning File	: C:\GCMSsolution\System\Tune1_default1.qgt
$EndIf$Modified by	: Mai Younis
Modified	: 03/09/2014 05:34:28 م

Method

==== Analytical Line 1 ====

IonSourceTemp	:250.00 °C
[MS Table]	
--Group 1 - Event 1--	
Start Time	:0.00min
End Time	:10.00min
ACQ Mode	:Scan
Event Time	:0.50sec
Scan Speed	:1428
Start m/z	:50.00
End m/z	:700.00
Electron Voltage	: 70 eV
Ionization Mode	: EI

C:\GCMSsolution\Data\Project1\3.QGD

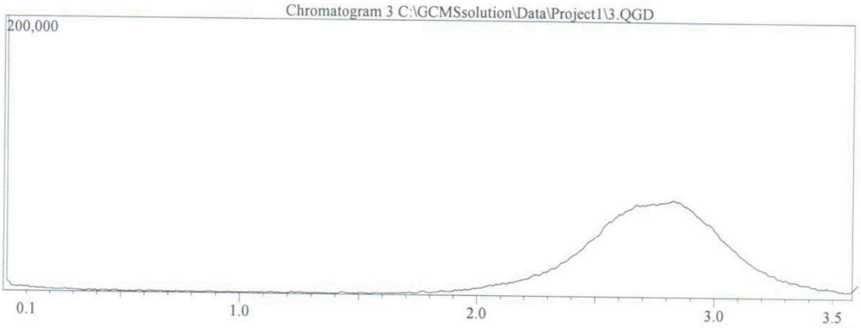

Spectrum

Line#:1 R.Time:2.8(Scan#:338)
MassPeaks:108
RawMode:Single 2.8(338) BasePeak:199(3345)
BG Mode:None Group 1 - Event 1

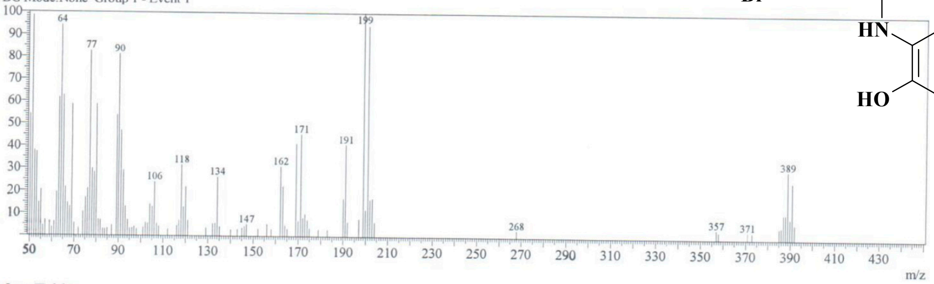

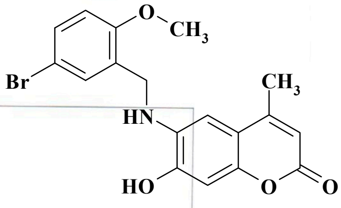

7

Mass Table
Line#:1 R.Time:2.8(Scan#:338)
MassPeaks:108
RawMode:Single 2.8(338) BasePeak:199(3345)
BG Mode:None Group 1 - Event 1

#	m/z	Abs. In	Rel. Int.	#	m/z	Abs. In	Rel. Int.	#	m/z	Abs. In	Rel. Int.
1	49.95	1811	54.14	4	52.95	1244	37.19	7	55.90	150	4.48
2	50.95	3296	98.54	5	53.95	494	14.77	8	56.90	231	6.91
3	52.00	1270	37.97	6	54.95	682	20.39	9	58.90	215	6.43

Figure S18. Mass Spectra of compound **7**.

12-Oct-14 20:51:08

Cairo University
Micro Analytical Center

DI Analysis
Shimadzu Qp-2010 Plus

Sample Information

Analyzed by	: A.GABR
Analyzed	: 12/10/2014 08:44:51 م
Sample Name	: 11
Sample ID	:
Customer Name	: رضوان الحجار - صيدليه حلوان
Data File	: C:\GCMSsolution\Data\Project1\11.QGD
Org Data File	: C:\GCMSsolution\Data\Project1\11.QGD
Method File	: C:\GCMSsolution\Data\Project1\A.GABR.qgm
Org Method File	: C:\GCMSsolution\Data\Project1\A.GABR.qgm
Report File	:
Tuning File	: C:\GCMSsolution\System\Tune1_default1.qgt
$EndIf$Modified by	: A.GABR
Modified	: 12/10/2014 08:49:59 م

Method

==== Analytical Line 1 ====

IonSourceTemp	:250.00 °C
[MS Table]	
--Group 1 - Event 1--	
Start Time	:0.00min
End Time	:10.00min
ACQ Mode	:Scan
Event Time	:0.50sec
Scan Speed	: 769
Start m/z	:50.00
End m/z	:400.00
Electron Voltage	: 70 eV
Ionization Mode	: EI

C:\GCMSsolution\Data\Project1\11.QGD

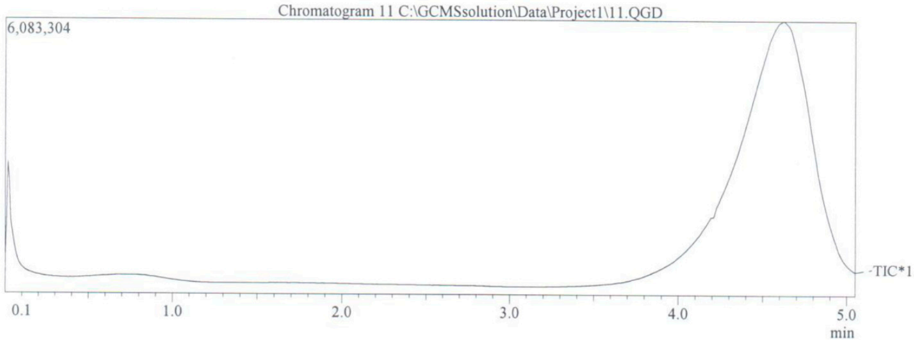

Spectrum

Line#:1 R.Time:5.0(Scan#:607)
MassPeaks:280
RawMode:Single 5.0(607) BasePeak:137(52372)
BG Mode:None Group 1 - Event 1

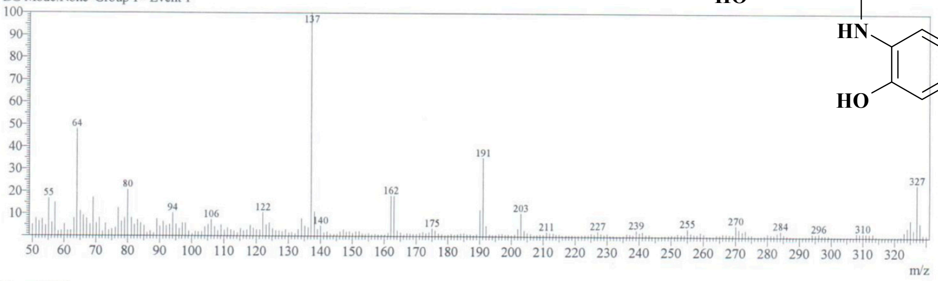

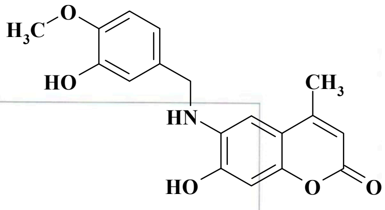

8

Mass Table
Line#:1 R.Time:5.0(Scan#:607)
MassPeaks:280
RawMode:Single 5.0(607) BasePeak:137(52372)
BG Mode:None Group 1 - Event 1

#	m/z	Abs. In	Rel. Int.	#	m/z	Abs. In	Rel. Int.	#	m/z	Abs. In	Rel. Int.
1	50.00	2620	5.00	4	53.05	3988	7.61	7	56.10	3070	5.86
2	51.05	4146	7.92	5	54.10	2430	4.64	8	57.10	7852	14.99
3	52.05	3405	6.50	6	55.05	8831	16.86	9	58.10	1050	2.00

Figure S19. Mass Spectra of compound **8**.

12-Oct-14 20:10:10

Cairo University
Micro Analytical Center

DI Analysis
Shimadzu Qp-2010 Plus

Sample Information

Analyzed by	: A.GABR
Analyzed	: 12/10/2014 08:03:12 م
Sample Name	: 7
Sample ID	:
Customer Name	: رضوان الحجار - صيدله حلوان
Data File	: C:\GCMSsolution\Data\Project1\7.QGD
Org Data File	: C:\GCMSsolution\Data\Project1\7.QGD
Method File	: C:\GCMSsolution\Data\Project1\A.GABR.qgm
Org Method File	: C:\GCMSsolution\Data\Project1\A.GABR.qgm
Report File	:
Tuning File	: C:\GCMSsolution\System\Tune1_default1.qgt
$EndIf$Modified by	: A.GABR
Modified	: 12/10/2014 08:07:07 م

Method

==== Analytical Line 1 ====

IonSourceTemp	:250.00 °C
[MS Table]	
--Group 1 - Event 1--	
Start Time	:0.00min
End Time	:10.00min
ACQ Mode	:Scan
Event Time	:0.50sec
Scan Speed	: 769
Start m/z	:50.00
End m/z	:400.00
Electron Voltage	: 70 eV
Ionization Mode	: EI

C:\GCMSsolution\Data\Project1\7.QGD

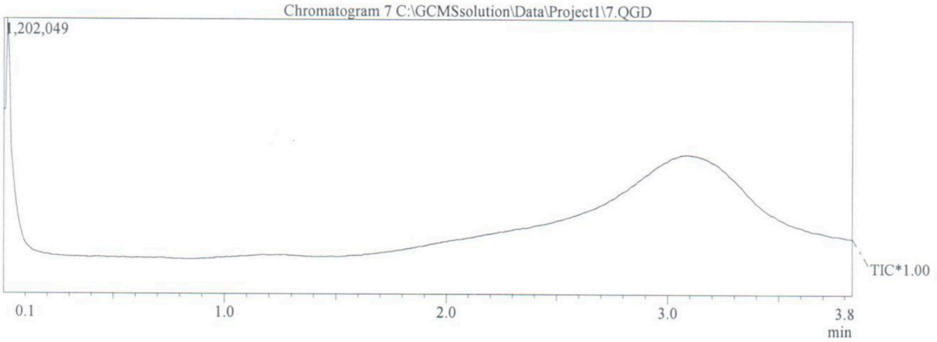

Spectrum

Line#:1 R.Time:2.2(Scan#:269)
MassPeaks:119
RawMode:Single 2.2(269) BasePeak:148(339)
BG Mode:2.3(273) Group 1 - Event 1

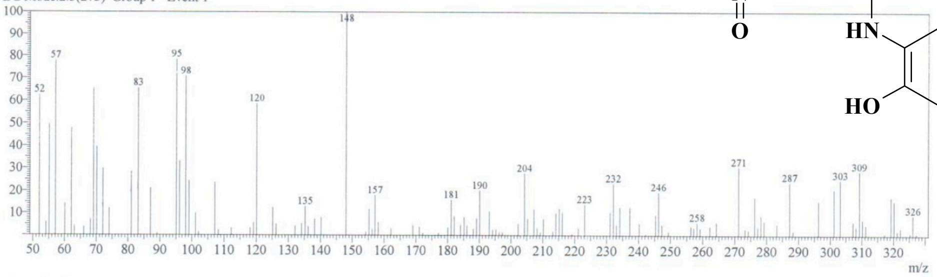

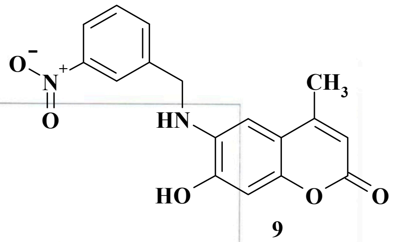

9

Mass Table
Line#:1 R.Time:2.2(Scan#:269)
MassPeaks:119
RawMode:Single 2.2(269) BasePeak:148(339)
BG Mode:2.3(273) Group 1 - Event 1

#	m/z	Abs. In	Rel. Int.	#	m/z	Abs. In	Rel. Int.	#	m/z	Abs. In	Rel. Int.
1	52.00	212	62.54	4	57.10	264	77.88	7	63.05	14	4.13
2	54.10	20	5.90	5	60.05	48	14.16	8	66.05	12	3.54
3	55.10	168	49.56	6	62.10	162	47.79	9	68.10	24	7.08

Figure S20. Mass Spectra of compound **9**.

12-Oct-14 20:32:11

Cairo University
Micro Analytical Center

DI Analysis
Shimadzu Qp-2010 Plus

Sample Information

Analyzed by	: A.GABR
Analyzed	: 12/10/2014 08:25:27 م
Sample Name	: 9
Sample ID	:
Customer Name	: رضوان الحجار - صيدله حلوان
Data File	: C:\GCMSsolution\Data\Project1\9.QGD
Org Data File	: C:\GCMSsolution\Data\Project1\9.QGD
Method File	: C:\GCMSsolution\Data\Project1\A.GABR.qgm
Org Method File	: C:\GCMSsolution\Data\Project1\A.GABR.qgm
Report File	:
Tuning File	: C:\GCMSsolution\System\Tune1_default1.qgt
$EndIf$Modified by	: A.GABR
Modified	: 12/10/2014 08:30:02 م

Method

==== Analytical Line 1 ====

IonSourceTemp	:250.00 °C
[MS Table]	
--Group 1 - Event 1--	
Start Time	:0.00min
End Time	:10.00min
ACQ Mode	:Scan
Event Time	:0.50sec
Scan Speed	: 769
Start m/z	:50.00
End m/z	:400.00
Electron Voltage	: 70 eV
Ionization Mode	: EI

C:\GCMSsolution\Data\Project1\9.QGD

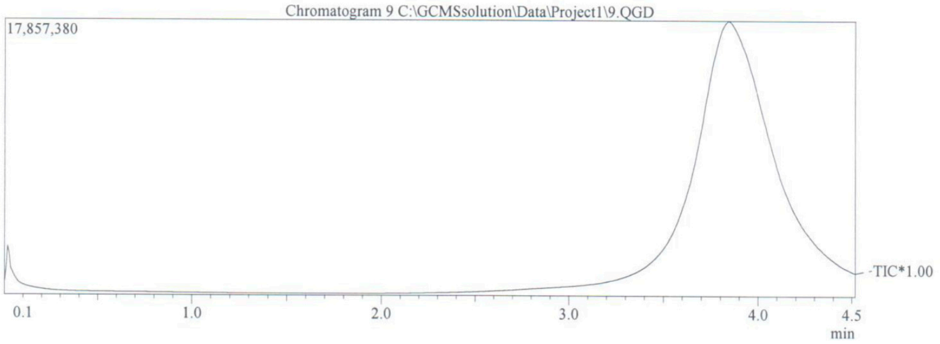

Spectrum

Line#:1 R.Time:4.3(Scan#:511)
MassPeaks:294
RawMode:Single 4.3(511) BasePeak:340(413426)
BG Mode:None Group 1 - Event 1

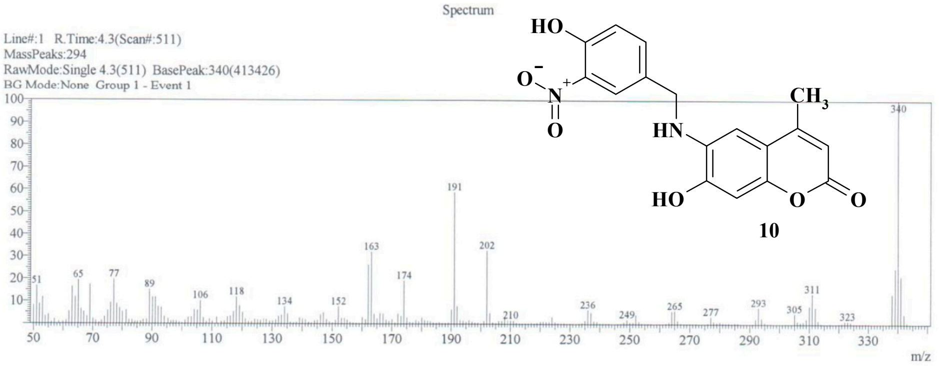

Mass Table
Line#:1 R.Time:4.3(Scan#:511)
MassPeaks:294
RawMode:Single 4.3(511) BasePeak:340(413426)
BG Mode:None Group 1 - Event 1

#	m/z	Abs. In	Rel. Int.	#	m/z	Abs. In	Rel. Int.	#	m/z	Abs. In	Rel. Int.
1	50.05	33677	8.15	4	53.05	48503	11.73	7	56.10	3318	0.80
2	51.05	69041	16.70	5	54.00	13337	3.23	8	57.10	8351	2.02
3	52.05	35890	8.68	6	55.05	16286	3.94	9	58.60	4522	1.09

1 / 3

Figure S21. Mass Spectra of compound **10**.

12-Oct-14 20:19:59

Cairo University
Micro Analytical Center

DI Analysis
Shimadzu Qp-2010 Plus

Sample Information

Analyzed by	: A.GABR
Analyzed	: 12/10/2014 08:12:17 م
Sample Name	: 8
Sample ID	:
Customer Name	: رضوان الحجار - صيدليه حلوان
Data File	: C:\GCMSsolution\Data\Project1\8.QGD
Org Data File	: C:\GCMSsolution\Data\Project1\8.QGD
Method File	: C:\GCMSsolution\Data\Project1\A.GABR.qgm
Org Method File	: C:\GCMSsolution\Data\Project1\A.GABR.qgm
Report File	:
Tuning File	: C:\GCMSsolution\System\Tune1_default1.qgt
$EndIf$Modified by	: A.GABR
Modified	: 12/10/2014 08:18:34 م

Method

==== Analytical Line 1 ====

IonSourceTemp	:250.00 °C
[MS Table]	
--Group 1 - Event 1--	
Start Time	:0.00min
End Time	:10.00min
ACQ Mode	:Scan
Event Time	:0.50sec
Scan Speed	: 769
Start m/z	:50.00
End m/z	:400.00
Electron Voltage	: 70 eV
Ionization Mode	: EI

C:\GCMSsolution\Data\Project1\8.QGD

Chromatogram 8 C:\GCMSsolution\Data\Project1\8.QGD

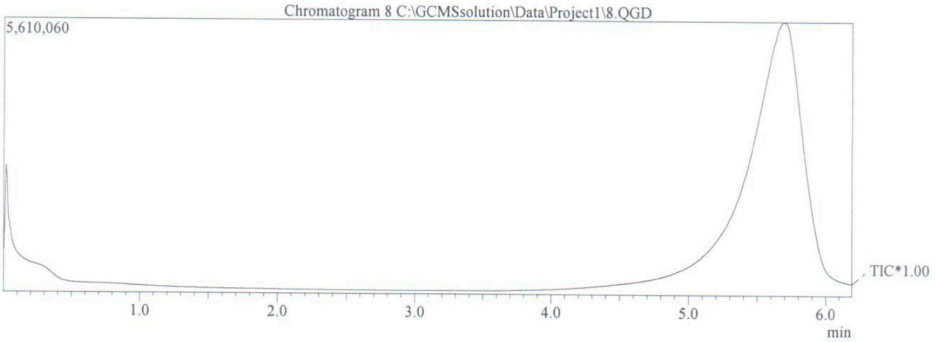

Spectrum

Line#:1 R.Time:4.3(Scan#:515)
MassPeaks:188
RawMode:Single 4.3(515) BasePeak:80(720)
BG Mode:4.2(510) Group 1 - Event 1

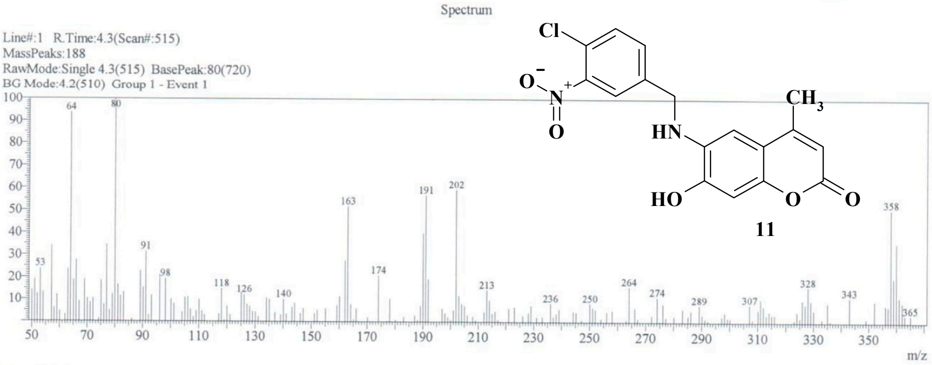

11

Mass Table
Line#:1 R.Time:4.3(Scan#:515)
MassPeaks:188
RawMode:Single 4.3(515) BasePeak:80(720)
BG Mode:4.2(510) Group 1 - Event 1

#	m/z	Abs. In	Rel. Int.	#	m/z	Abs. In	Rel. Int.	#	m/z	Abs. In	Rel. Int.
1	50.00	102	14.17	4	53.05	171	23.75	7	58.05	44	6.11
2	51.05	137	19.03	5	54.10	96	13.33	8	59.05	86	11.94
3	52.00	89	12.36	6	57.10	245	34.03	9	60.05	33	4.58

1 / 2

Figure S22. Mass Spectra of compound **11**.

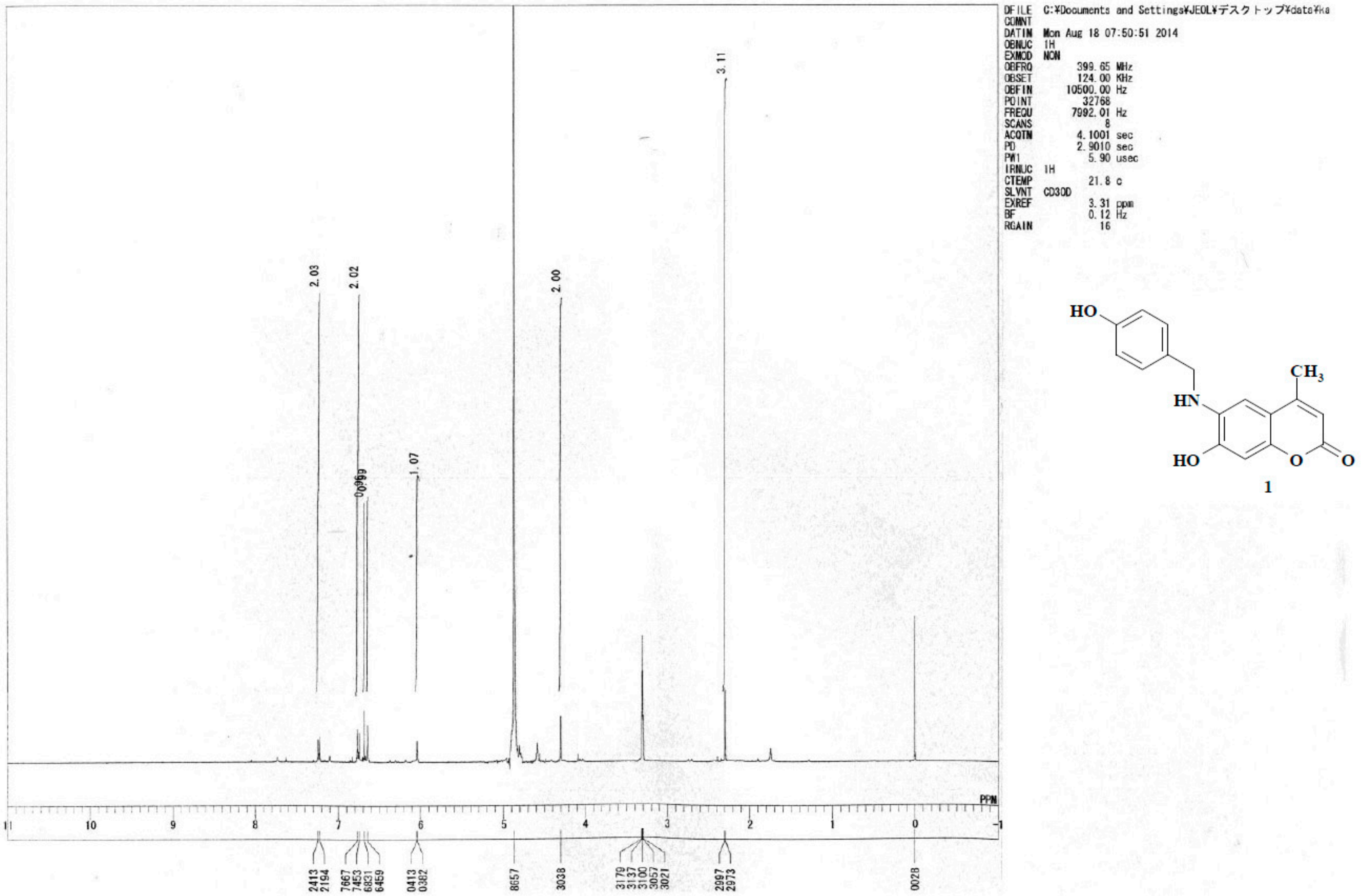

Figure S23. ^{1}H-NMR of compound **1**.

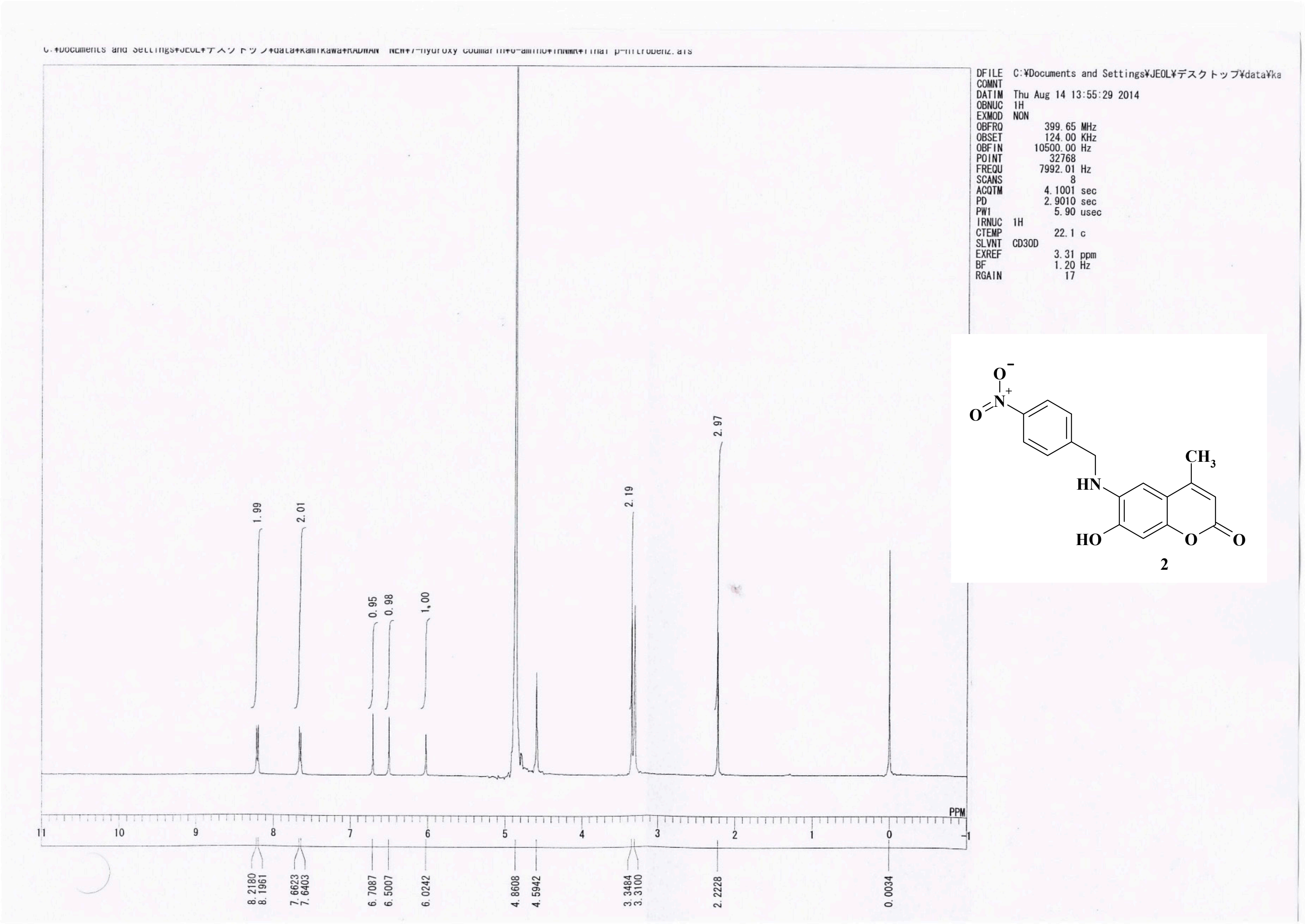

Figure S24. ^{1}H-NMR of compound **2**.

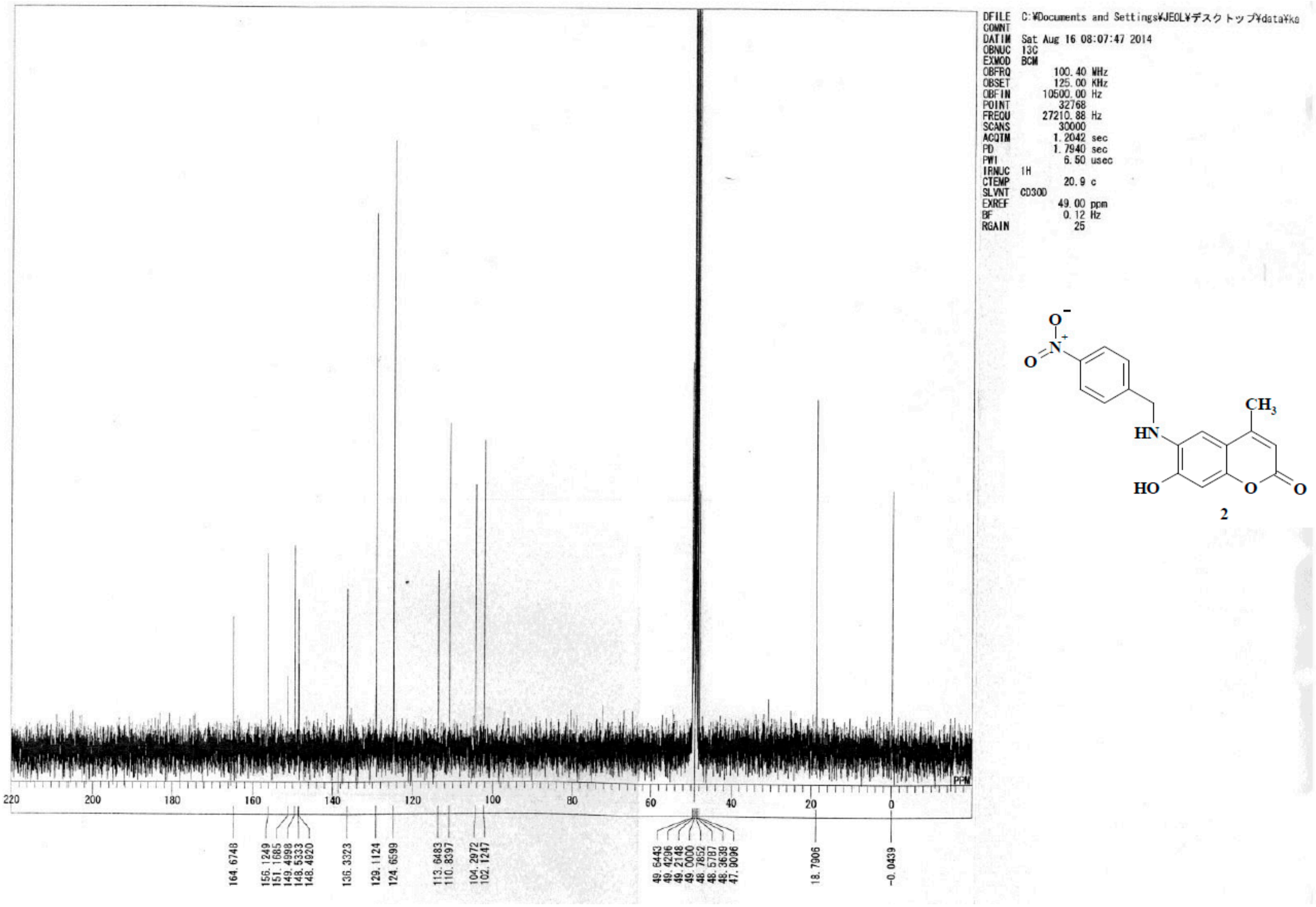

Figure S25. ^{13}C-NMR of compound **2**.

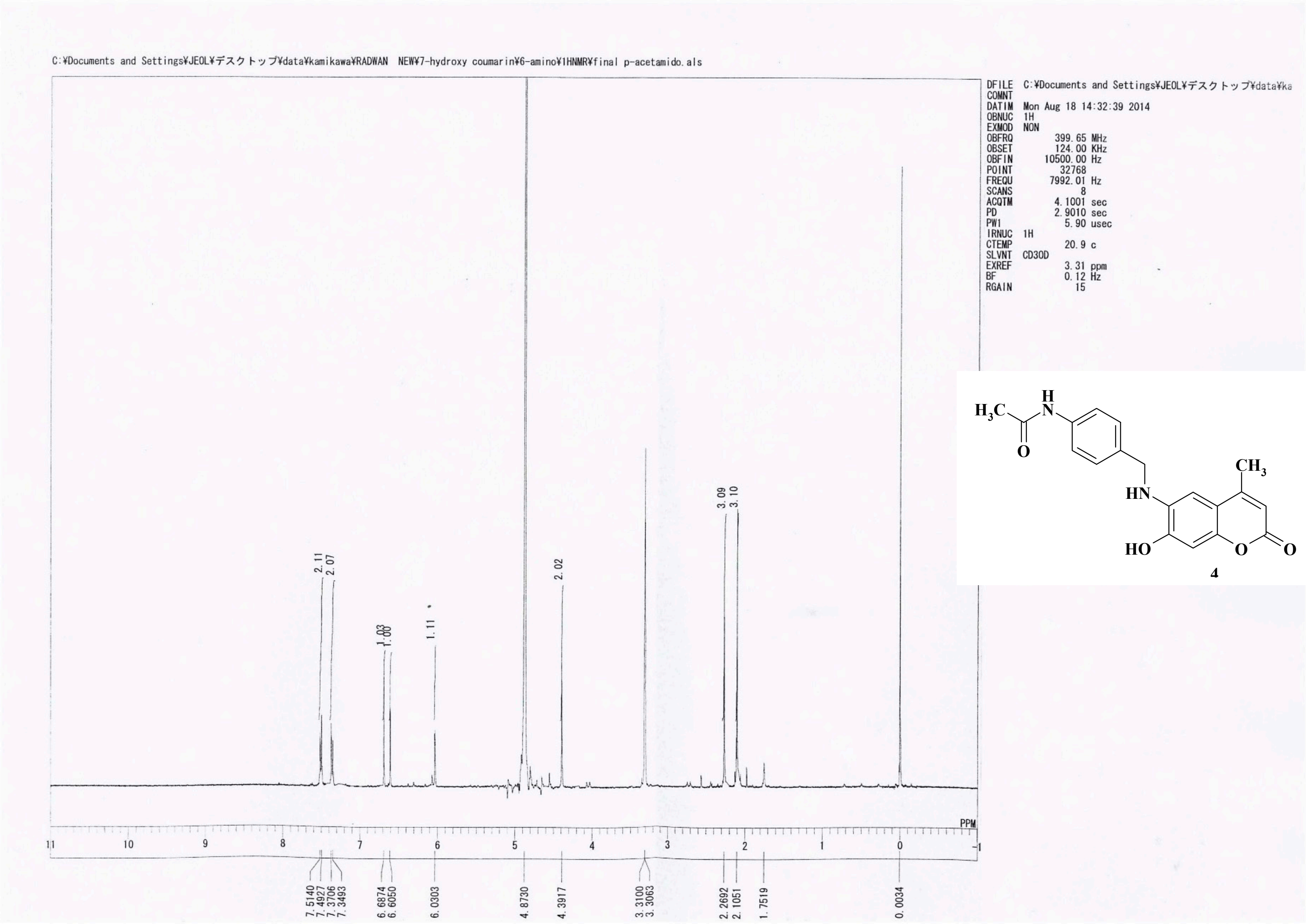

Figure S26. ^{1}H-NMR of compound **4**.

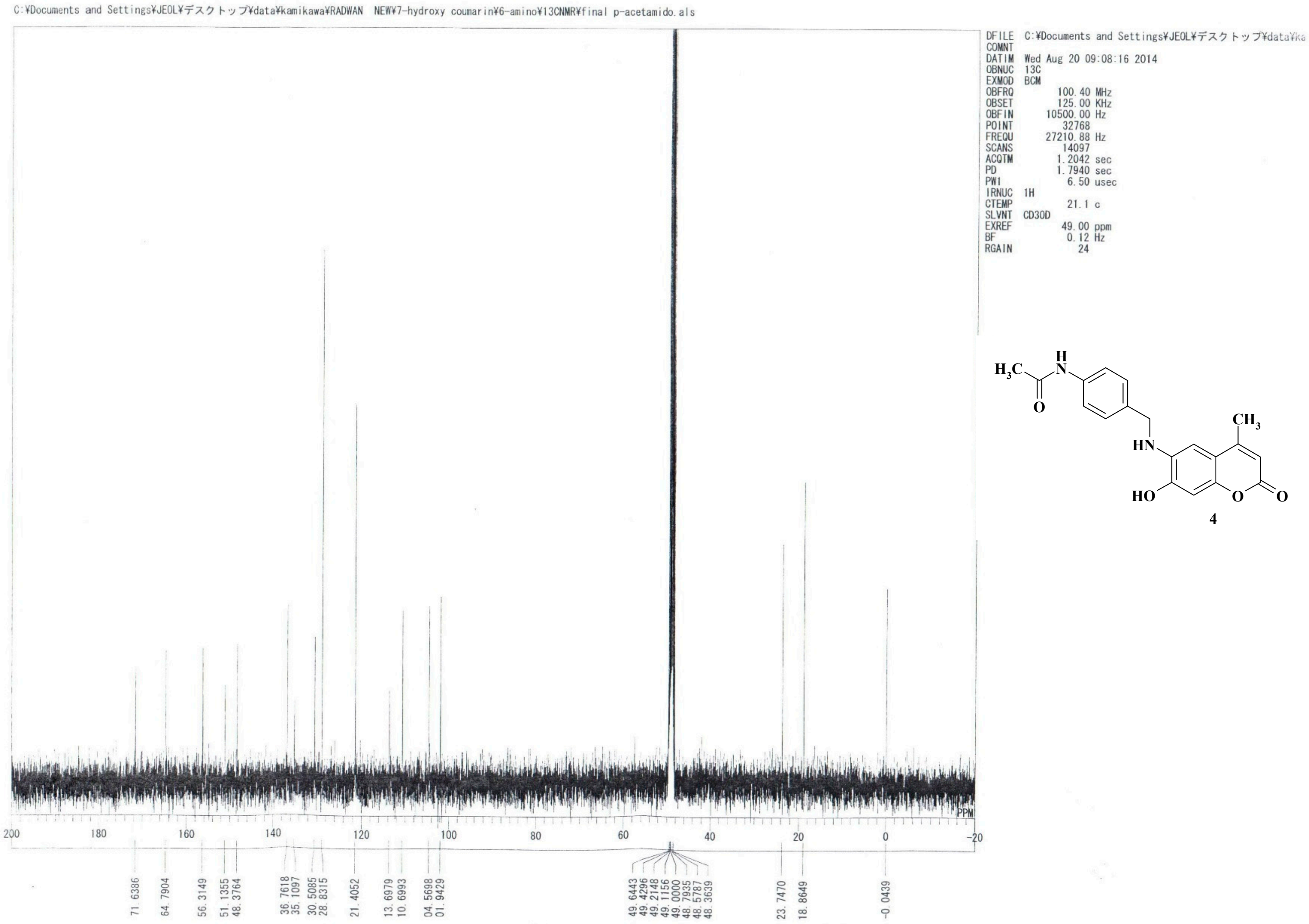

4

Figure S27. ^{13}C-NMR of compound **4**.

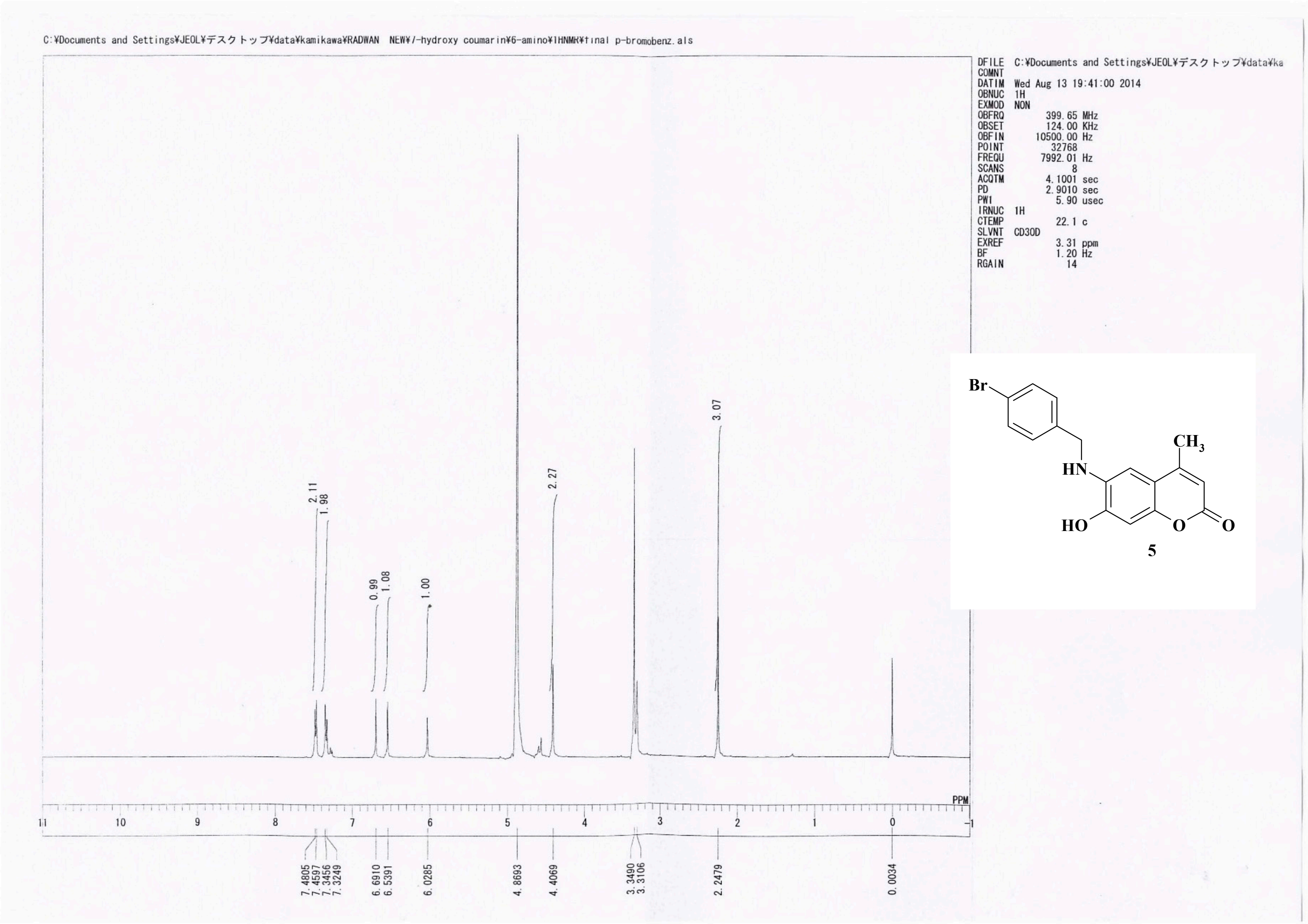

Figure S28. ^{1}H-NMR of compound **5**.

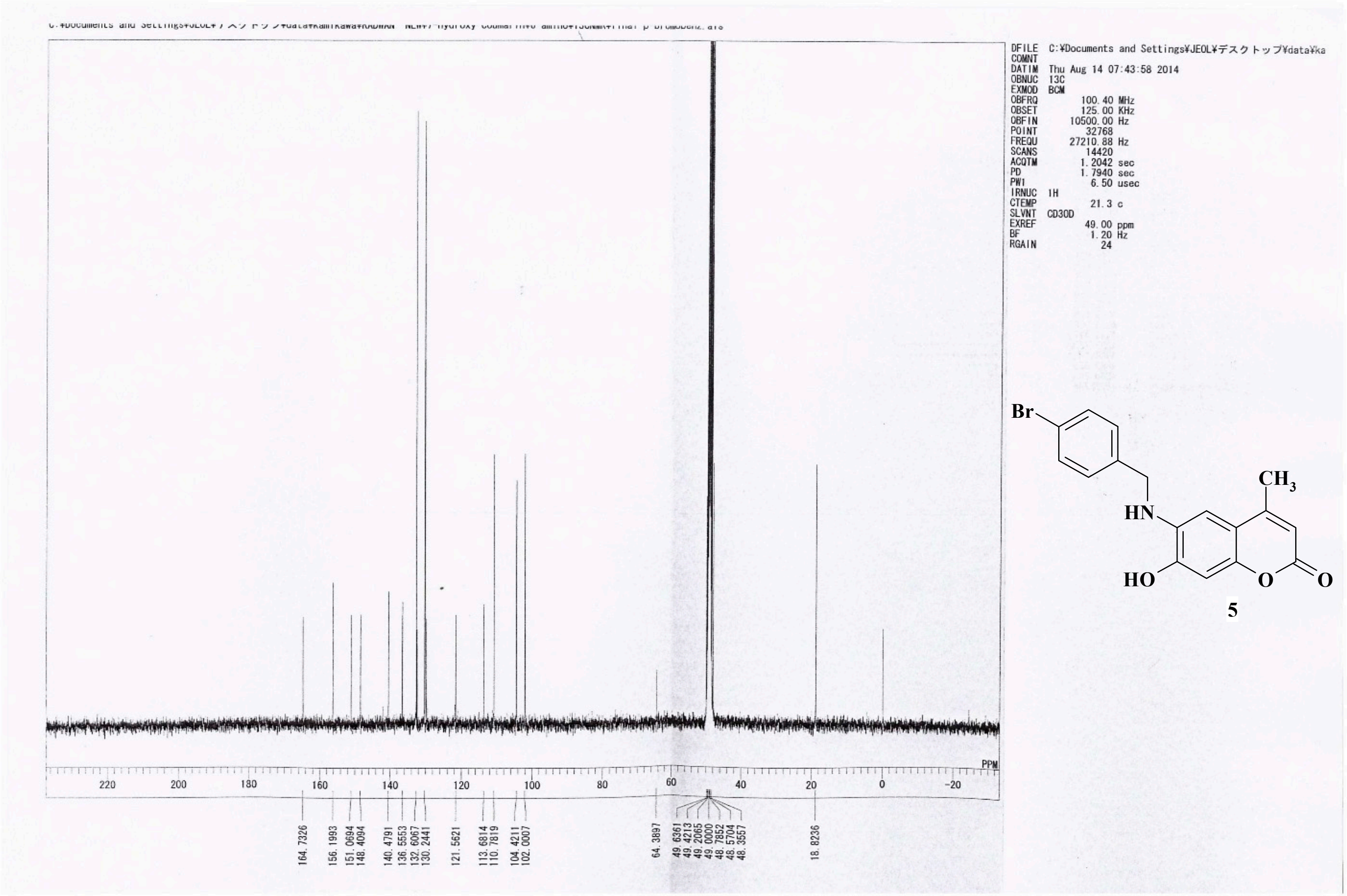

5

Figure S29. ^{13}C-NMR of compound **5**.

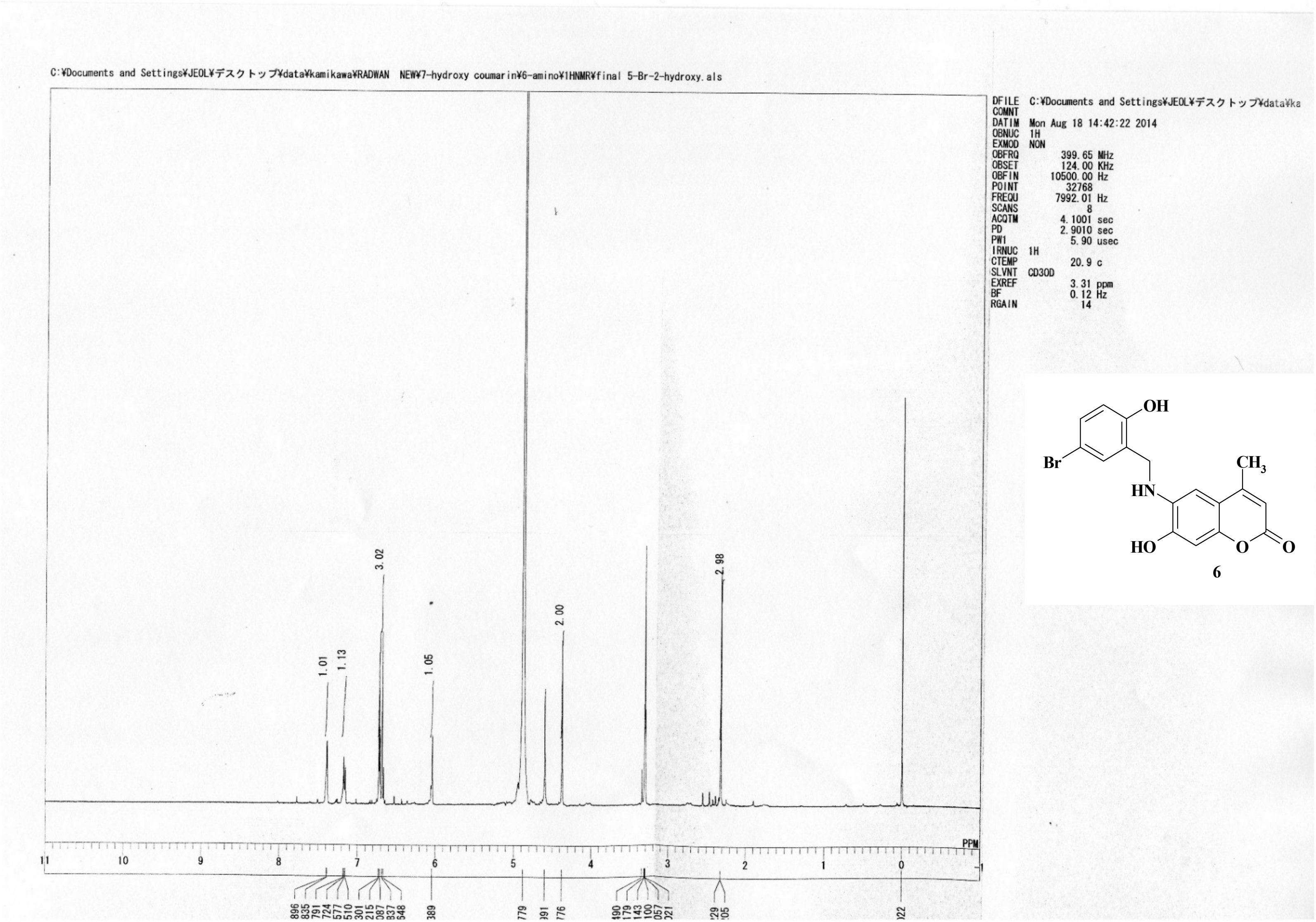

Figure S30. ^{1}H-NMR of compound **6**.

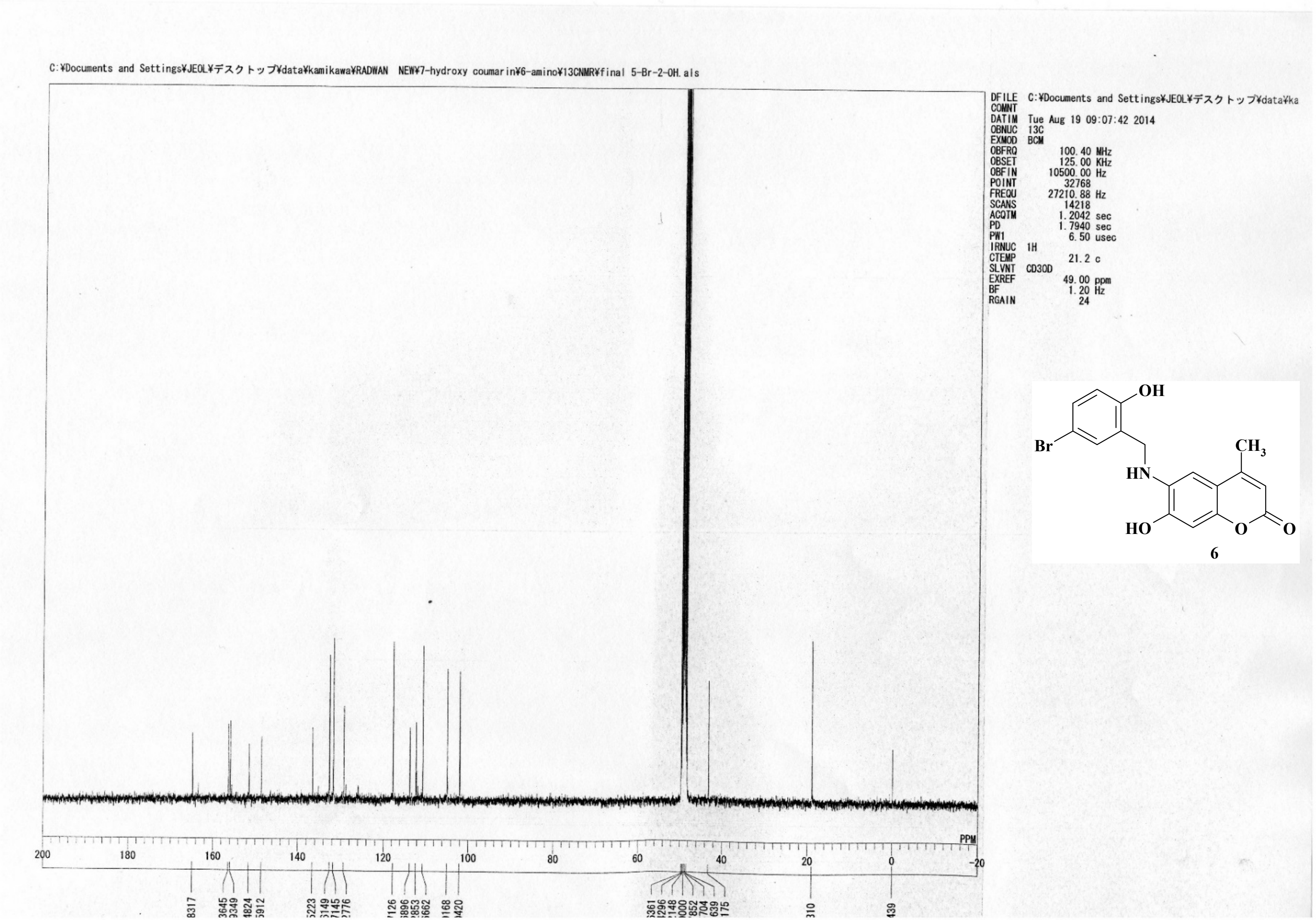

6

Figure S31. ^{13}C-NMR of compound **6**.

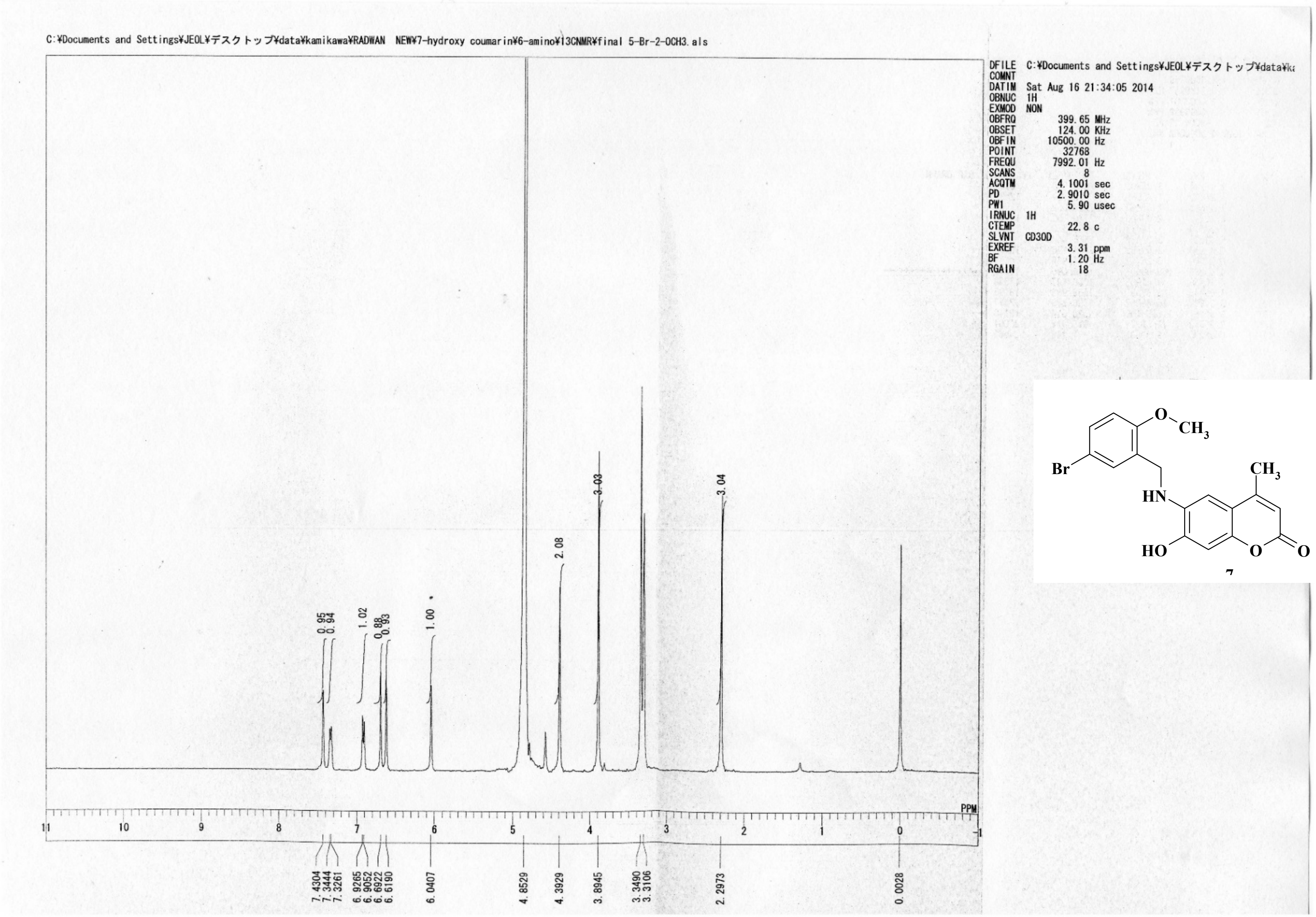

Figure S32. ^{1}H-NMR of compound **7**.

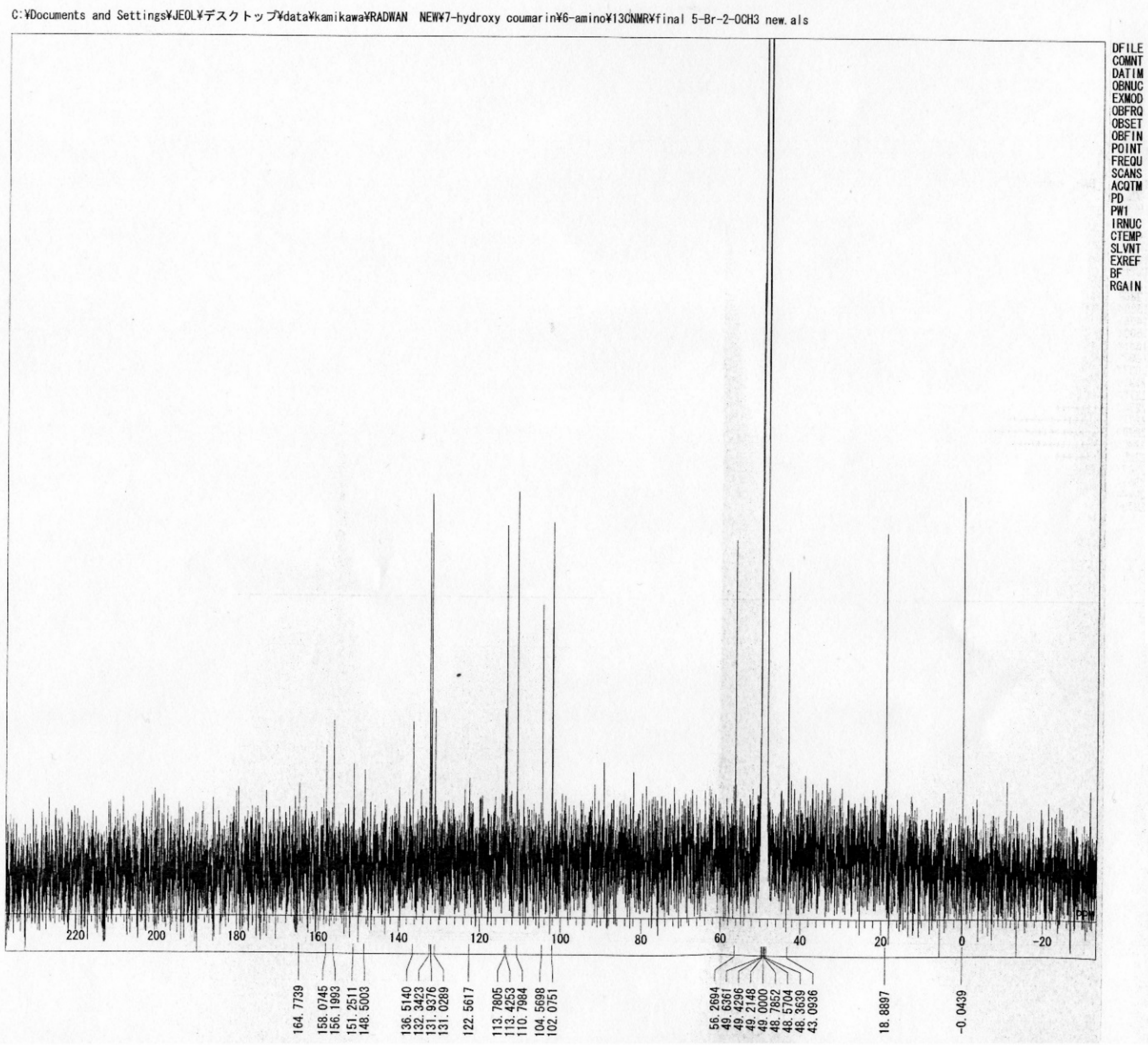

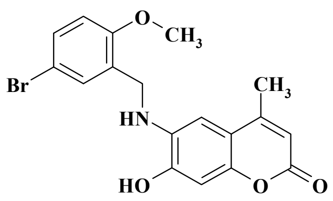

7

Figure S33. ^{13}C-NMR of compound **7**.

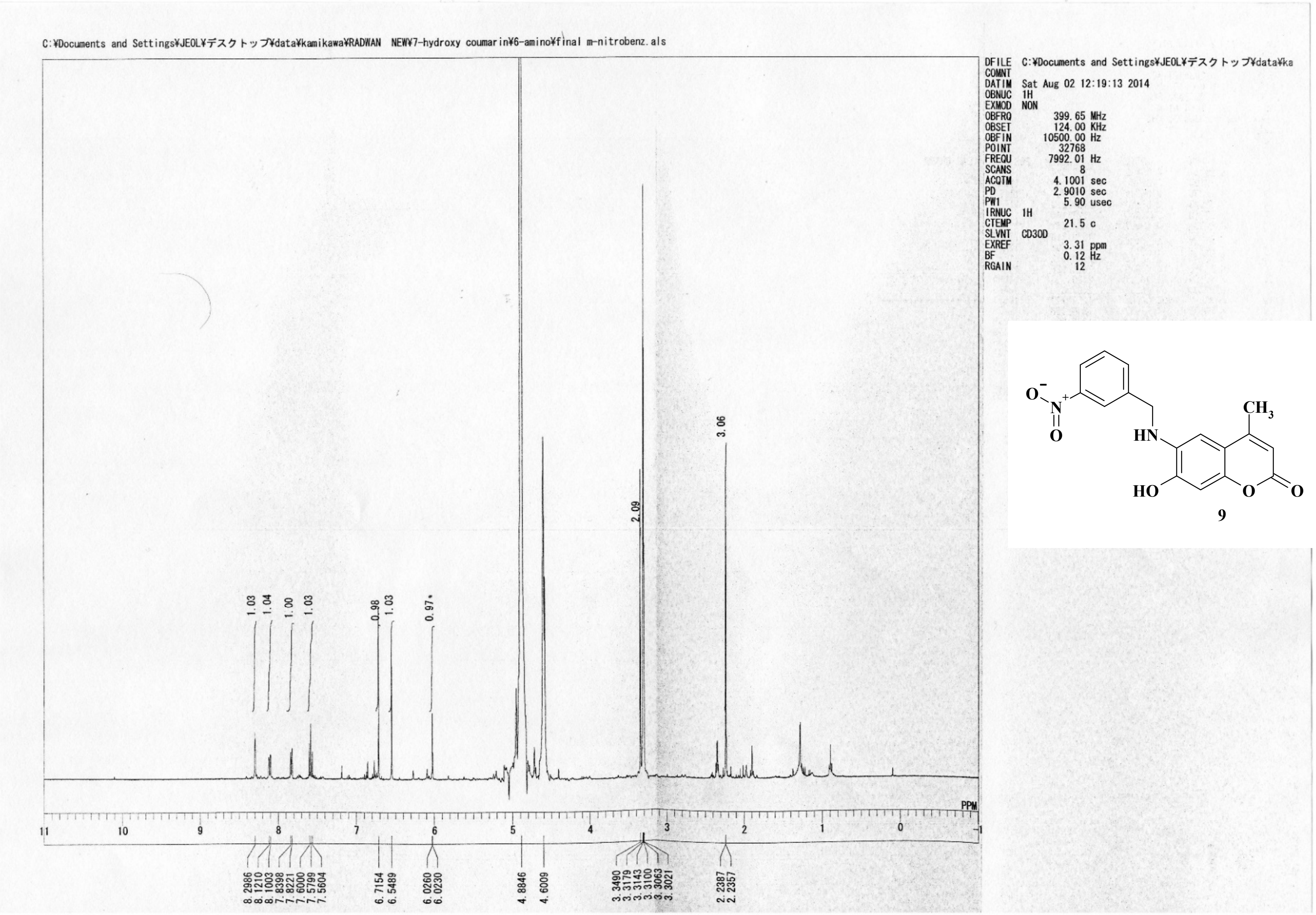

Figure S34. ^{1}H-NMR of compound **9**.

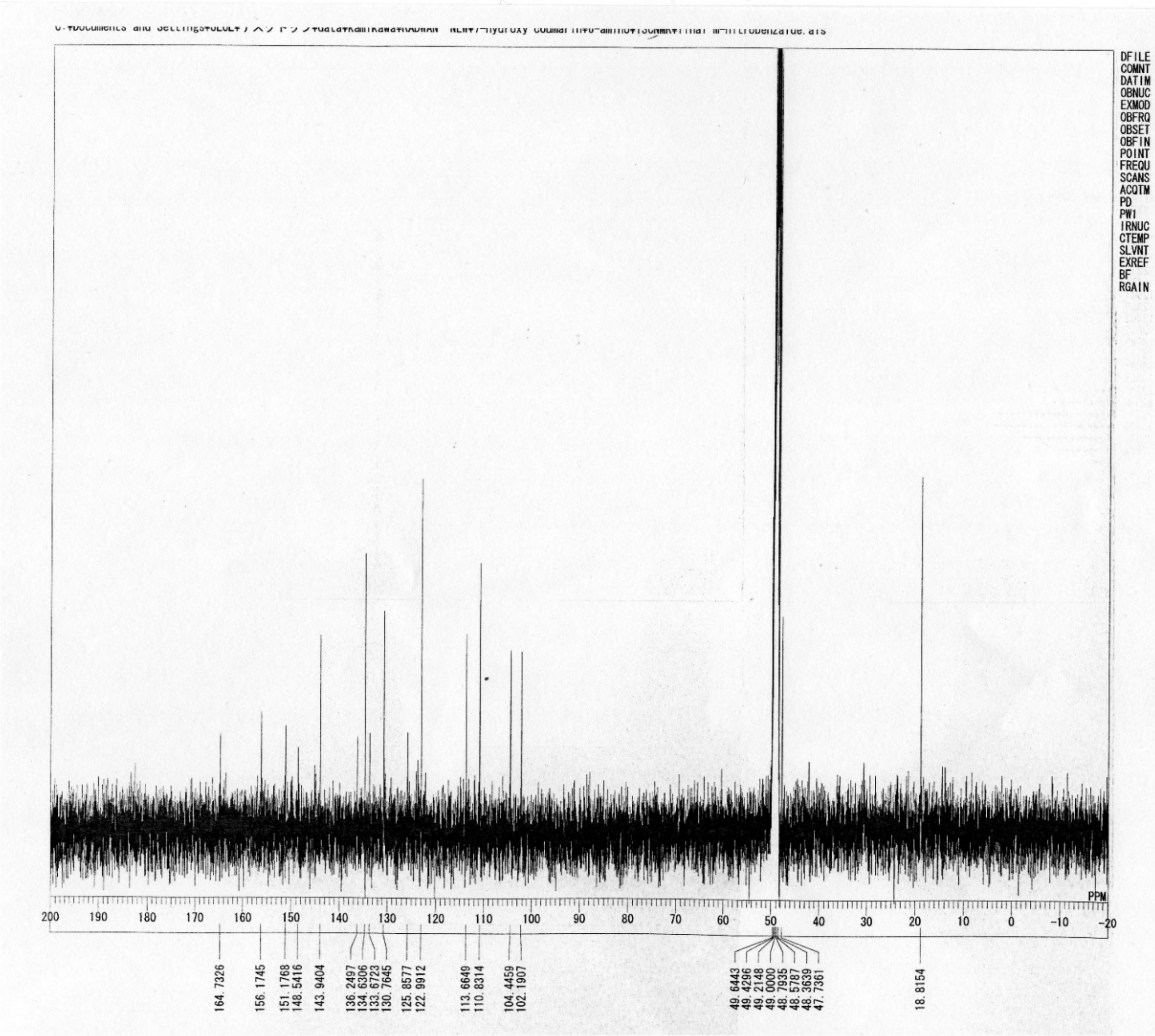

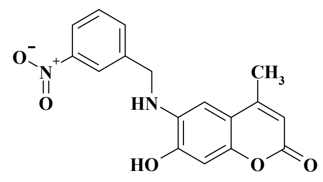
9

Figure S35. ^{13}C-NMR of compound **9**.
